# Two-year intermittent exposure of a multiwalled carbon nanotube by intratracheal instillation induces lung tumors and pleural mesotheliomas in F344 rats

**DOI:** 10.1186/s12989-022-00478-7

**Published:** 2022-05-19

**Authors:** Motoki Hojo, Ai Maeno, Yoshimitsu Sakamoto, Aya Ohnuki, Yukie Tada, Yukio Yamamoto, Kiyomi Ikushima, Ryota Inaba, Jin Suzuki, Yuhji Taquahashi, Satoshi Yokota, Norihiro Kobayashi, Makoto Ohnishi, Yuko Goto, Takamasa Numano, Hiroyuki Tsuda, David B. Alexander, Jun Kanno, Akihiko Hirose, Akiko Inomata, Dai Nakae

**Affiliations:** 1 Department of Pharmaceutical and Environmental Sciences, Tokyo Metropolitan Institute of Public Health, 3-24-1 Hyakunincho, Shinjuku, Tokyo 169-0073 Japan; 2 Center for Biological Safety and Research, National Institute of Health Sciences, Kanagawa, Japan; 3 Japan Bioassay Research Center, Japan Organization of Occupational Health and Safety, Kanagawa, Japan; 4 DIMS Institute of Medical Science, Aichi, Japan; 5 Nanotoxicology Project, Nagoya City University, Aichi, Japan; 6 Animal Medical Course, Department of Medical Sports, Faculty of Health Care and Medical Sports, Teikyo Heisei University, 4-1 Uruido-Minami, Ichihara, Chiba, 290-0193 Japan; 7 Department of Nutritional Science and Food Safety, Faculty of Applied Biosciences, Tokyo University of Agriculture, Tokyo, Japan

**Keywords:** MWCNT, Carcinogenicity, Intratracheal instillation, Lung tumors, Pleural mesothelioma, Lung burden

## Abstract

**Background:**

A mounting number of studies have been documenting the carcinogenic potential of multiwalled carbon nanotubes (MWCNTs); however, only a few studies have evaluated the pulmonary carcinogenicity of MWCNTs in vivo. A 2-year inhalation study demonstrated that MWNT-7, a widely used MWCNT, was a pulmonary carcinogen in rats. In another 2-year study, rats administered MWNT-7 by intratracheal instillation at the beginning of the experimental period developed pleural mesotheliomas but not lung tumors. To obtain data more comparable with rats exposed to MWNT-7 by inhalation, we administered MWNT-7 to F344 rats by intratracheal instillation once every 4-weeks over the course of 2 years at 0, 0.125, and 0.5 mg/kg body weight, allowing lung burdens of MWNT-7 to increase over the entire experimental period, similar to the inhalation study.

**Results:**

Absolute and relative lung weights were significantly elevated in both MWNT-7-treated groups. Dose- and time-dependent toxic effects in the lung and pleura, such as inflammatory, fibrotic, and hyperplastic lesions, were found in both treated groups. The incidences of lung carcinomas, lung adenomas, and pleural mesotheliomas were significantly increased in the high-dose group compared with the control group. The pleural mesotheliomas developed mainly at the mediastinum. No MWNT-7-related neoplastic lesions were noted in the other organs. Cytological and biochemical parameters of the bronchoalveolar lavage fluid (BALF) were elevated in both treated groups. The lung burden of MWNT-7 was dose- and time-dependent, and at the terminal necropsy, the average value was 0.9 and 3.6 mg/lung in the low-dose and high-dose groups, respectively. The number of fibers in the pleural cavity was also dose- and time-dependent.

**Conclusions:**

Repeated administration of MWNT-7 by intratracheal instillation over the 2 years indicates that MWNT-7 is carcinogenic to both the lung and pleura of rats, which differs from the results of the 2 carcinogenicity tests by inhalation or intratracheal instillation.

**Supplementary Information:**

The online version contains supplementary material available at 10.1186/s12989-022-00478-7.

## Background

For the last two decades, multiwalled carbon nanotubes (MWCNT) were one of the most successful materials in nanotechnology because of their remarkable mechanical, thermal, chemical, and electrical properties. Applications of MWCNTs have expanded from electrochemical and biomedical fields to areas such as automobile materials, asphalt road pavement, and electrical distribution systems. Accordingly, the discharge of significant waste which can be emitted into the air is expected. There are numerous types of MWCNT with different physico-chemical properties, but, particle toxicologists have first focused on a group of MWCNT fibers with needle-like structure because their shape and physical and chemical durability may be similar to asbestos. Early intraperitoneal injection studies showed that a thick, long, and straight fiber, MWNT-7, induced peritoneal mesothelioma in rodents [1, 2], while a relatively thin and short fiber did not [3]. Initial studies using inhalation exposure to MWCNTs, a more relevant exposure route for humans than intraperitoneal injection, found that MWNT-7 caused pulmonary toxicity [4, 5]. Studies using intratracheal instillation and pharyngeal aspiration, which are more cost-effective hazard assessment methods compared to inhalation exposure, also found that MWNT-7 caused pulmonary toxicity [6–10]. The toxicological findings of both types of studies included acute inflammation with infiltration of neutrophils, aggregations of fiber-engulfing macrophages, fibrosis, microgranuloma formation, and reactive hyperplasias of the alveolar epithelial cells.

A landmark study was published by Kasai et al*.* in 2016: this study exposed rats by whole-body inhalation to MWNT-7 and demonstrated that MWNT-7 was a pulmonary carcinogen [11]. They exposed 50 male and female rats to 3 different concentrations of MWNT-7 for 6 h/day, 5 days/week for 2 years and clearly showed dose-dependent carcinogenicity.

Whole-body inhalation studies, however, require specialized facilities and only a few of these facilities exist. MWCNTs are highly heterogenous materials, and in vitro and in vivo studies conducted over the last decade have shown that their toxicities depend on their physico-chemical features [12–17]. Thus, in addition to MWNT-7, which was classified as a Group 2B carcinogen by the International Agency for Research on Cancer in 2011, carcinogenicities of other varieties of MWCNTs should be examined. However, implementation of long-term tests using inhalation systems specific to each test material may be unrealistic. Tsuda et al*.* have established a protocol for 2-year studies of carbon nanotubes using intratracheal administration of the test material with a micro-sprayer several times at the beginning of the study period followed by a subsequent 2-year observation period without treatment: this protocol was named intra-Tracheal Intra-Pulmonary Spraying (TIPS) [18, 19]. Using TIPS, Suzui et al. showed that MWCNT-N, an MWCNT similar to MWNT-7, induced lung tumors and pleural mesotheliomas [20]; Numano et al*.* showed that MWNT-7 induced pleural mesotheliomas [21]; and Saleh et al*.* showed that MWCNT-B, a thin and tangled fiber, induced lung tumors [22]. Intratracheal instillation is suitable for hazard identification and characterization without the use of special facilities, and consequently can be used to screen a large number of test materials, which currently is not possible using whole body inhalation testing. However, intratracheal instillation studies differ from inhalation studies in regards to lung burden (herein, lung burden refers to the amount of the test materials retained in the lung) during the experimental period. Continuous exposure by inhalation to insoluble materials results in increasing lung burden throughout the experimental period, while intratracheal instillation administration protocols such as TIPS results in maximal lung burden at the beginning of the study period.

Both exposure level and duration can affect the outcomes of carcinogenicity tests. In the inhalation study by Kasai et al., lung burden increased from 0 to 1.8 mg/lung in male rats exposed to the highest levels of MWNT-7. These rats developed lung tumors and simple mesothelial hyperplasia, but not pleural mesothelioma [11]. In contrast, in the intratracheal instillation study by Numano et al., rats were instilled with 1.5 mg MWNT-7 at the beginning of the study period and 18 of 19 MWNT-7-treated rats developed malignant pleural mesothelioma, one rat died from a pituitary tumor [21]. The lack of mesothelioma development in the inhalation study could have been due to the relatively low levels of fibers that translocated into the pleural cavity combined with the relatively short duration of exposure of the mesothelium to the fibers [11, 23]. Lack of significant induction of lung tumors in the instillation study was thought to be due to the early deaths caused by the development of malignant mesothelioma [21]. Thus, the differences in the results of the 2-year inhalation and instillation studies could be attributed to the difference in the temporal pattern of MWNT-7 lung burden in the two studies. Implementation of a new intratracheal instillation protocol in which the temporal change in the lung burden is similar to the inhalation study could be one of the ways to validate this proposal.

The present study evaluated the carcinogenicity of MWNT-7 administered by intratracheal instillation to rats using an administration protocol in which MWNT-7 lung burden gradually increased over an experimental period of 2 years. We administered two dosages of MWNT-7 (0.125 and 0.5 mg/kg body weight) to male F344 rats once every 4 weeks over the course of 2 years. Fibers in the lung tissue and pleural lavage fluid were measured at 26, 52, and 104 weeks. The administration protocol used in this study resulted in total MWNT-7 lung burdens of 0.9 mg/lung and 3.6 mg/lung in the low and high dose groups at 2 years. Notably, rats exposed to the higher levels of MWNT-7 developed both pulmonary carcinomas and pleural mesotheliomas.

## Results

### Characterization of the MWCNT

**Fig. 1:**
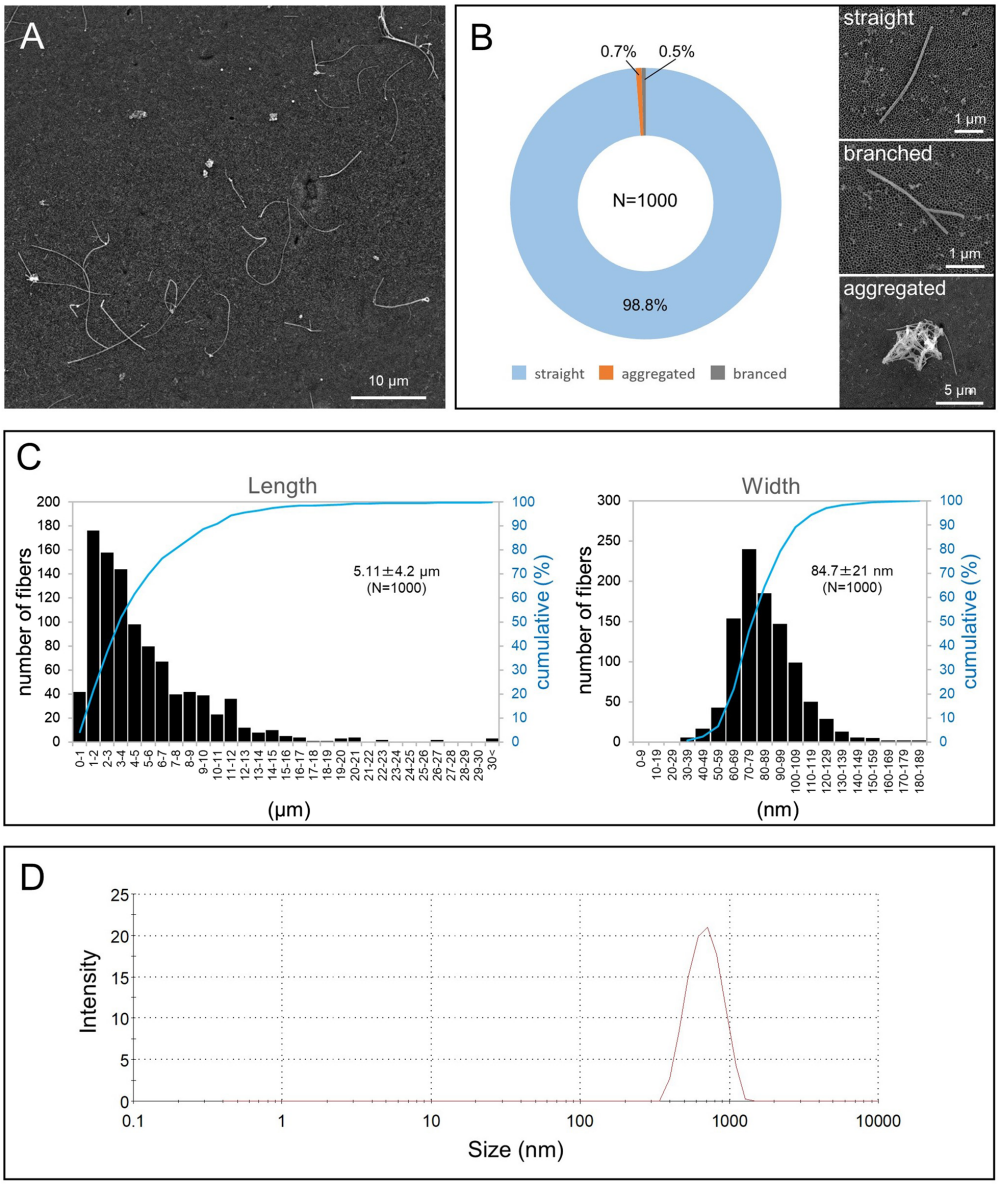
Characterization of the test material. **A** SEM image of Taquann-MWNT-7. **B** Morphological classification of the individual fibers. The straight fibers account for 98.8% of all examined fibers (left). Representative images of the 3 structures (right), **C** Length and width distribution of MWNT-7. **D** DLS analysis. The peak of the hydrodynamic diameter is 669 nm

In this study, to obtain well-dispersed single fibers, the bulk MWNT-7 was processed by the Taquann method [24]. A typical scanning electron microscope (SEM) image of the Taquann-MWNT-7 fibers in the suspension media used for administration is shown in Fig. 1A. Almost all fibers had a single, straight structure; only a few fibers were branched (0.5%) or aggregated (0.7%) (Fig. 1B). The average length and width of the MWNT-7 fibers were 5.11 µm and 84.7 nm, respectively (Fig. 1C). One gram of MWNT-7 corresponded to 1.83 × 10^13^ fibers.

Dynamic light scattering (DLS) measurement indicated that the secondary particle diameter of the MWNT-7 in the suspension was 669 nm (Fig. 1D).

### Interim sacrifice schedule and macroscopic findings

For the interim analyses, 4 or 5 rats from each group were sacrificed at weeks 26 and 52. The lungs and mediastinal lymph nodes of the MWNT-7-treated rats had gray to black colored MWCNT depositions. The degree of darkness at the lung surface increased with the dose and time. No macroscopic lesions were found.

### Body weights and survival

**Fig. 2:**
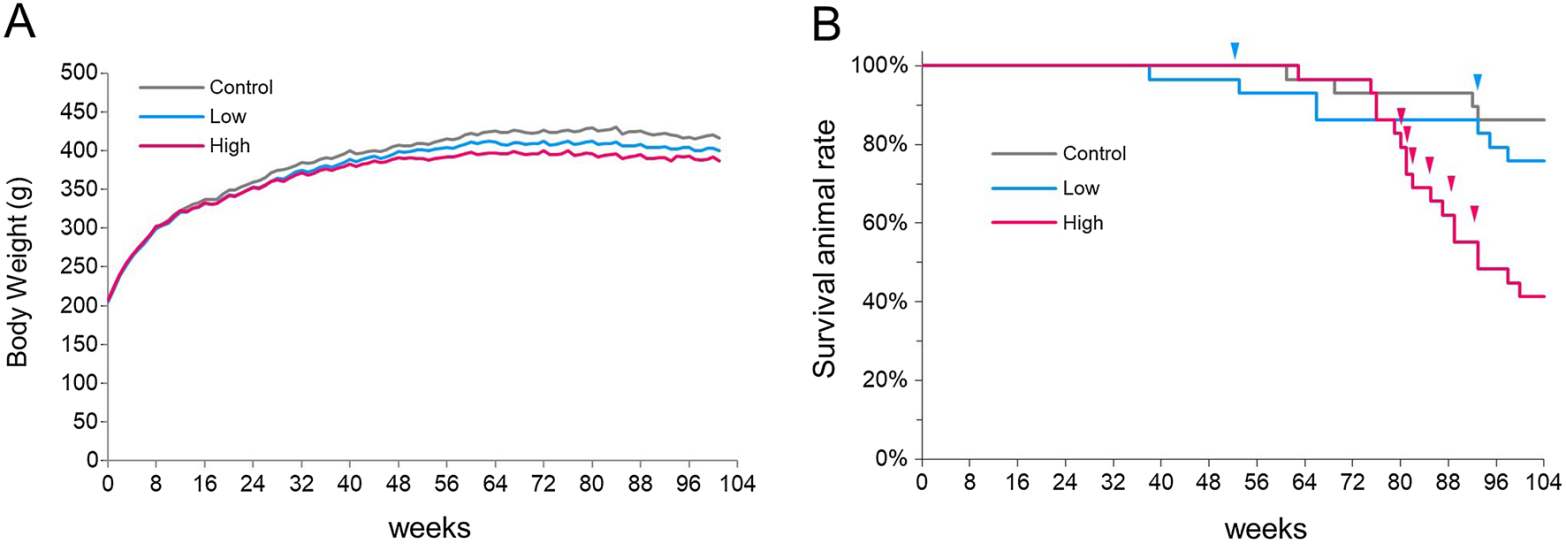
Growth and survival rate of the vehicle and MWNT-7-treated groups. **A** Body weight curves. Slight growth retardation occurred in both MWCNT-treated groups. Significant reduction was observed from week 28 to week 104 in the high-dose group and from week 68 to week 96 in the low-dose group. **B** Survival rates by Kaplan–Meier plots. A significant difference was detected between the control group and the high-dose group. Arrowheads show the pleural mesothelioma-related deaths

Body weight curves are shown in Fig. 2A. Compared with the control group, slight growth retardation was found in the MWNT-7-treated groups, and significant declines in body weight were observed in the high-dose group from week 28 to week 104 and in the low-dose group from week 68 to week 96.

Before the terminal necropsy, 6 animals died during administration of the test materials or from undetermined causes, and 1 animal died from a pituitary tumor and was not suitable for histological evaluation; these 7 animals were excluded from the study (see Additional File. 1: Table S1 footnotes). As shown in a Kaplan–Meier survival plot (Fig. 2B), a significant decrease in the average survival time was observed in the high-dose group compared with the control group. The average survival time for each group is shown in Table 1. The first rat that died from pleural mesothelioma was found at week 53 in the low-dose group. Including this case, a total of 2 and 7 rats died or were euthanized before the end of the study because of pleural mesothelioma-related pathological conditions in the low-dose group and high-dose group, respectively (Fig. 2B and Additional File. 1: highlighted by red letters in Table S2). There were 5, 5, and 9 non-treatment-related deaths in the control, low-dose, and high-dose groups, respectively (Additional File. 1: Table S2).

**Table 1:** Survival period, body weights, and lung weights in rats at week 104

	Control	Low-dose	High-dose
Number of animals examined	30	29	28
Survival animal number (rate; %)	25 (83.3)	22 (75.9)	12 (41.4)
Average survival time (weeks)	99.7	96.5	92.8
Body weight (g)	417.9 ± 34	399.1 ± 30	384.6 ± 17*
Absolute lung weight (g)	1.32 ± 0.08	1.92 ± 0.1*	2.91 ± 0.3*
Relative lung weight (g/100 g body weight)	0.31 ± 0.03	0.48 ± 0.05*	0.76 ± 0.06*

Weights: Mean ± SD

*Significantly different from Control (by Dunnett’s test)

### Macroscopic findings at the terminal necropsy

**Fig. 3:**
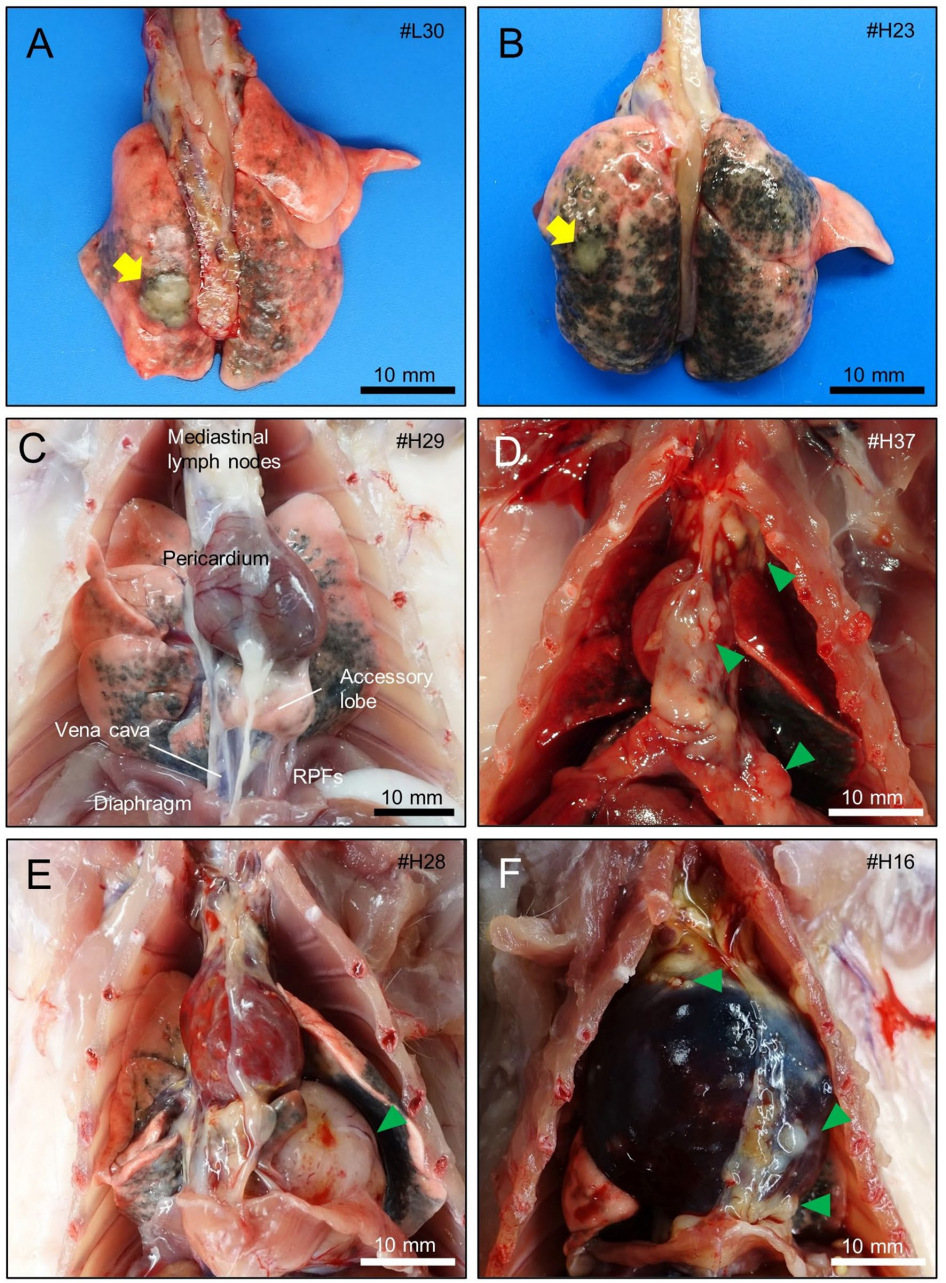
Gross appearances of the lung and thoracic viscera of MWNT-7-treated rats. **A** Whitish and yellow nodule (yellow arrow, adenoma) on the surface of the lung of a rat in the low-dose group. The rat was sacrificed at week 104. **B** White nodule (yellow arrow, adenoma) on the surface of the lung of a rat in the high-dose group. The rat was sacrificed due to the progression of a neck tumor (hemangiosarcoma) at week 89. **C** Appearance of the mediastinum of a rat that had no proliferative lesions. Retrocardiac pleural folds (RPFs) extend from the pericardium to the diaphragm, and enwrap the accessory lobe. **D**, **E**, **F** Malignant pleural mesotheliomas found in the high-dose group (sacrificed at weeks 81, 93, and 81). Multiple small or large nodules of the mesothelioma are seen on the surface of the serosa of the mediastinum (green arrowheads). **F** Rat with a cardiac tamponade evidenced by the enlarged pericardium containing bloody fluid

Rats that died or were sacrificed before the end of the study at week 104 are listed in Additional File. 1: Table S2: these animals were included in the study. The remaining 69 animals underwent terminal necropsy at week 104 (Additional File. 1: Table S1). The lung surfaces of the MWNT-7-treated rats appeared gray to black, and the tone was darker in the high-dose group than in the low-dose group. Two and 4 animals had small yellowish-white nodules on the surface of the lung in the low-dose and high-dose groups (Fig. 3A and B). Two and 8 rats (low-dose and high-dose groups, respectively) had small to large nodules and/or thick sheets on the surface of the lung, chest wall, pericardium, diaphragm, and retrocardiac pleural folds (RPFs) (Fig. 3C–F). The RPF is a pair of serous membranes, located at the caudal region of the thorax, connecting the pericardium with the ventral chest wall and the diaphragm (Fig. 3C, Additional File. 2: Figs. S1A–1D) [25, 26]. The right side of the RPF is usually called the “*plica venae cavae*” because the *vena cava* runs along it in the thoracic cavity. Three animals with mesothelioma in the high-dose group developed severe cardiac tamponade and/or bloody fluid accumulated in the pleural cavity (Fig. 3F).

### Body weights, organ weights and hematological analysis at the terminal sacrifice at week 104

At week 104, a slight but significant reduction was noted in the body weights of rats in the high-dose group compared with the control group (approximately 8% lower; Table 1). Absolute and relative lung weights were significantly elevated in both treated groups (Table 1). Neither treatment-related increases nor decreases in weights were found in other organs (Additional File. 1: Table S3). Hematological analysis revealed a significant increase in the eosinophil count in the high-dose group compared with the control group (Additional File. 1: Table S4).

### Microscopic findings

**Fig. 4:**
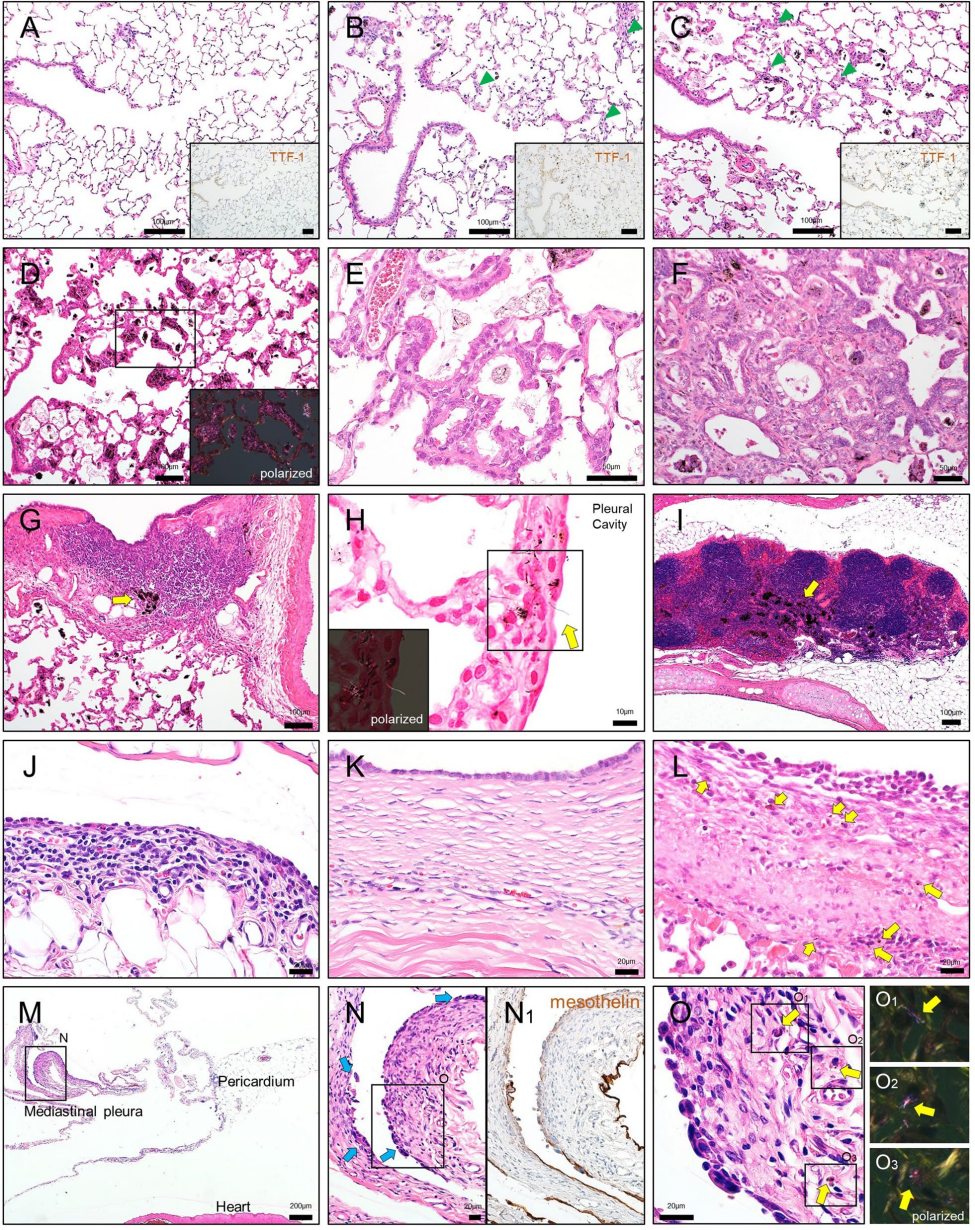
Microscopic findings of non-neoplastic lesions in the lung and pleura of MWNT-7-treated rats. **A**–**C** Representative histological images of the lung in rats of the control (**A**), low-dose (**B**), and high-dose (**C)** groups at week 26. Foci of reactive alveolar hyperplasia are scattered (arrowheads). The proliferation of the alveolar epithelium is evidenced by an increase in the immunopositivity of the nuclei for TTF-1 in the MWNT-7 treated groups (insets). **D** Marked MWCNT depositions in the alveolar regions of the lung of a rat in the high-dose group. Inset: a polarized light microscope image. **E** Bronchiolization (low-dose group). **F** Atypical hyperplasia (high-dose group). Epithelial cells form irregular structures (often glandular structures) with fibrous connective tissue proliferations and MWCNT depositions. **G** MWCNT depositions in a BALT (arrow; high-dose group). **H** MWCNT fiber piercing the visceral pleura (arrow) (high-dose group). Kernechtrot staining. Inset: polarized light microscope image. **I** MWCNT depositions in a mediastinal lymph node (arrow; high-dose group). **J** Thickened mesothelium of the RPF (high-dose group). Enlarged mesothelial cells and inflammatory cells such as lymphocytes and eosinophils are seen. **K** Focal fibrosis of the diaphragm (low-dose group). The lesion is lined by enlarged and ciliated mesothelial cells. **L** Thickened visceral pleura with a fibrotic change (high-dose group). Monocytes, enlarged mesothelial cells, and MWCNT-engulfing macrophages (arrows) are notable. **M** Thickening of the mediastinal pleura containing hyperplastic lesions (high-dose group). **N** Higher magnification of (**M**). Several focal hyperplasias can be seen as protrusions (arrows). (N_1_) Immunostaining for mesothelin. **O** Higher magnification of (N). Layered or bulging mesothelial cells with atypia are underlain by a thickened submesothelial area. MWCNT fibers phagocyted by macrophages are distributed (arrows). Insets: polarized light microscope images. All images were obtained from animals sacrificed at week 104, except (**A**), (**B**), and (**C**)

The results of the histopathological examination of non-neoplastic lesions at the interim and terminal necropsies are shown in Table 2 and Fig. 4.

**Table 2:** Histological analysis of non-neoplastic lesions in the lung and pleura

Time-point		Week 26			Week 52			Week 104		
Group		Control	Low-dose	High-dose	Control	Low-dose	High-dose	Control	Low-dose	High-dose
Number of rats examined		5	5	5	5	4	4	27	26	24
Lung	Accumulation, alveolar macrophage	1	5*	5*	0	4*	4*	6	26*	24*
		[0.05]	[1.34*]	[1.86*]	[0]	[1.74*]	[2.63*]	[0.08]	[2.55*]	[2.84*]
	Focal fibrosis, alveolar wall	0	5*	5*	0	4*	4*	0	26*	24*
		[0]	[1.42*]	[1.86*]	[0]	[1.65*]	[2.56*]	[0]	[2.59*]	[3.19*]
	Granulomatous change	0	3	5*	0	4*	4*	0	26*	24*
		[0]	[0.10]	[1.12*]	[0]	[0.86*]	[1.71*]	[0]	[1.31*]	[2.52*]
	Reactive alveolar hyperplasia	0	5*	5*	0	4*	4*	0	26*	24*
		[0]	[0.61*]	[1.39*]	[0]	[1.60*]	[2.04*]	[0]	[2.43*]	[2.60*]
	Bronchiolar hyperplaisa (bronchiolization)	0	4*4^a^	0	0	1	4*	1	26*	24*
		[0]	[0.80*]	[0.20]	[0]	[0.25]	[1.00*]	[0.04]	[2.19*]	[2.92*]
	Bronchioloalveolar hyperplasia	0	1	0	2	0	0	6	16*	22*
		[0]	[0.20]	[0]	[0.40]	[0]	[0]	[0.46]	[1.67*]	[2.50*]
	Atypical hyperplasia	0	0	0	0	0	0	0	4	10*
		[0]	[0]	[0]	[0]	[0]	[0]	[0]	[0.43*]	[0.92*]
Pleura	Infiltration of inflammatory cells	0	3	4*	0	4*	4*	0	24*	23*
		[0]	[0.80]	[1.00*]	[0]	[0.75*]	[1.75*]	[0]	[1.52*]	[1.92*]
	Focal fibrosis	0	2	4*	0	4*	4*	0	26*	24*
		[0]	[0.60]	[1.40*]	[0]	[1.25*]	[1.75*]	[0]	[2.38*]	[2.75*]
	Mesothelial hyperplasia	0	0	0	0	0	0	0	9*	17*
		[0]	[0]	[0]	[0]	[0]	[0]	[0]	[0.62*]	[1.42*]

Values show the number of animals bearing the lesion

Values in square brackets are the average severity grades in all examined animals; $\left( {\Sigma \left( {{\text{grade}} \times {\text{number}}\;{\text{of}}\;{\text{animals}}\;{\text{with}}\;{\text{grade}}} \right)} \right) \div {\text{number}}\;{\text{of}}\;{\text{animals}}\;{\text{examined}}$

Grade: 0, no/minimal change; 1, slight; 2, moderate; 3, marked; 4, severe

The column “week 104” includes animals that died or were euthanized before the termination

Animals bearing large granular lymphocytic leukemia (LGL) were excluded (3, 3, and 4 animals in the control, low-dose, and high-dose groups, respectively)

*Significantly different from a corresponding control group (incidence, Fischer's exact test; severity score, Steel's test)

At the interim sacrifices, MWNT-7-treated rats had chronic inflammatory lesions throughout the lung parenchyma. Large numbers of MWCNT fibers were deposited in alveolar walls and macrophages in the alveoli, while some singlet or bundled fibers were found in the alveolar space and subpleura. At both 26 and 52 weeks, there was an accumulation of the macrophages engulfing MWNCT fibers, fibrosis in the alveolar walls, and granulomatous changes (Table 2). In association with MWCNT deposition, proliferation of type II alveolar cells was observed (Fig. 4A–C). Focal infiltrations of neutrophils and lymphocytes were present in the MWNT-7-treated groups. In the pleural mesothelium, fibrotic changes and enlarged mesothelial cells were seen. The incidence and severity of pulmonary and pleural lesions tended to increase in a dose- and time-dependent manner (Table 2). No tumors were found in the lung, pleura, or other organs at weeks 26 or 52.

At the terminal necropsy, the types of non-neoplastic findings in the lungs were similar to those found at the interim necropsies; however, the morphometrical analyses revealed that the degree of each lesion was more severe (Table 2). For instance, there was a marked accumulation of macrophages engulfing MWCNTs, and fractured alveolar macrophages were often observed within the alveoli (Fig. 4D). The size and number of granulomas were increased. Induction of hyperplastic lesions was elevated in the MWNT-7-treated groups (Table 2 and Fig. 4E and F). Atypical hyperplasias, precancerous proliferative lesions [11, 27], were only found at week 104 (Fig. 4F and Table 2). MWCNT fibers were widely distributed throughout the parenchyma, *e.g.*, in alveolar macrophages, thickened alveolar walls, granulomas, and bronchiolar associated lymphatic tissues (BALTs) (Fig. 4D and G). Fibers were also observed in the subpleura. A careful histological search revealed that a substantial number of fibers appeared to be piercing the visceral pleura (Figs. 4H and Additional File. 2: S2). MWCNT fibers were also deposited in the mediastinal lymph nodes; most of these fibers were engulfed by macrophages (Fig. 4I). In the thoracic cavity, focal pleural thickenings with edematous and/or fibrotic changes were observed (Fig. 4J–L). Mesothelial cells were slightly enlarged on the surface of the lesions (Fig. 4J). In some cases, the reactivity of mesothelial cells was prominent (Fig. 4K and L). Mesothelial hyperplasia was observed as a protrusion of 2- or 3-layered atypical mesothelial cells accompanied by proliferation of submesothelial cells, in which singlet or macrophage-laden fibers were scattered (Fig. 4L and O). The enlarged mesothelial cells and mesothelial hyperplasia were frequently seen in the mediastinum (Fig. 4M–O).

**Fig. 5:**
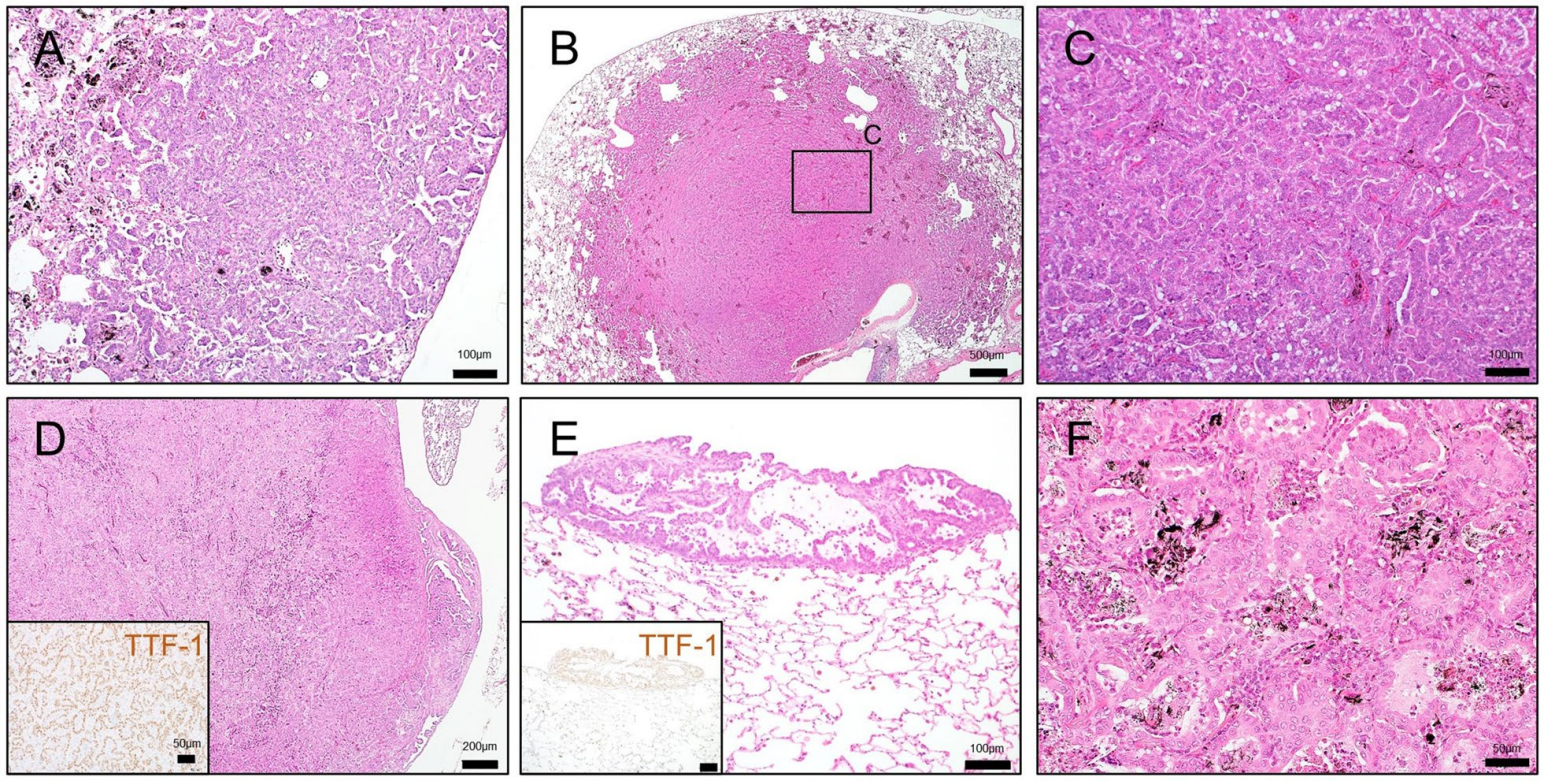
Lung tumors found in MWNT-7-treated rats. **A** Bronchiolo-alveolar adenoma in a rat of the high-dose group. Underlying alveolar architecture is obscure, and MWCNT-deposited areas appear to be compressed to the peripheral regions. **B** Bronchiolo-alveolar adenocarcinoma in a rat of the high-dose group. Tumor shows nodular growth (approximately 5 mm in diameter) and invasion of the surrounding tissues. **C** Higher magnification of (**B**). The tumor exhibits a glandular growth pattern with structural atypia. **D** Bronchiolo-alveolar adenocarcinoma in a rat of the low-dose group. Inset: immunostaining for TTF-1. The tumor shows aggressive invasion toward the visceral pleura at the right-posterior lobe, a primary site. **E** Translocated cells found on the surface of the right-anterior lobe. Inset: immunostaining for TTF-1. **F** Nonkeratinizing epithelioma (synonymous with benign nonkeratinizing squamous cell tumor) in a rat of the high-dose group. The alveolar space is filled with squamous cells with round to oval nuclei and fine granular eosinophilic cytoplasm

The results of the histopathological examination of neoplastic lesions in the lung and pleura are shown in Table 3. The incidences of adenocarcinomas, adenomas, and total lung tumors in the high-dose group were significantly increased compared with those in the control group. One rat in the control group developed an adenocarcinoma. Most lung tumors were single tumors, but 1 rat in the high-dose group had multiple tumors (2 adenomas and 1 adenocarcinoma). In the adenomas, tumor cells formed solid or papillary structures, making a sharp demarcation from the surrounding tissue (Fig. 5A). Neoplastic cells of the adenocarcinoma exhibited structural atypia, destroyed the normal alveolar structure, and invaded the interstitial tissue and vessels (Fig. 5B and C). In one rat in the low-dose group, an adenocarcinoma expanded outside of the lung parenchyma, and malignant cells translocated to other lobes (Fig. 5D and E). In addition, 1 rat in the high-dose group had a nonkeratinizing epithelioma (Fig. 5F).

**Table 3:** Incidence of lung tumors and malignant pleural mesothelioma

	Control	Low-dose	High-dose
Number of rats examined	30	29	28
Lung			
Bronchiolo-alveolar adenocarcinoma (%)	1 (3.3)	2 (6.9)	6* (21.4)
Bronchiolo-alveolar adenoma (%)	0 (0)	1 (3.5)	4* (14.3)
Non-keratinizing epithelioma (%)	0 (0)	0 (0)	1 (3.6)
Total tumors (%)	1 (3.3)	3 (10.3)	11* (39.3)
Pleura			
Malignant pleural mesothelioma (%)	0	4 (13.8)	12* (42.9)

Values show the number of animals bearing the tumor

Values in parentheses are the percentage of tumor incidences; 100 × number of animals with tumor ÷ number of animals examined

*Significantly different from Control (by Fisher's exact test)

**Fig. 6:**
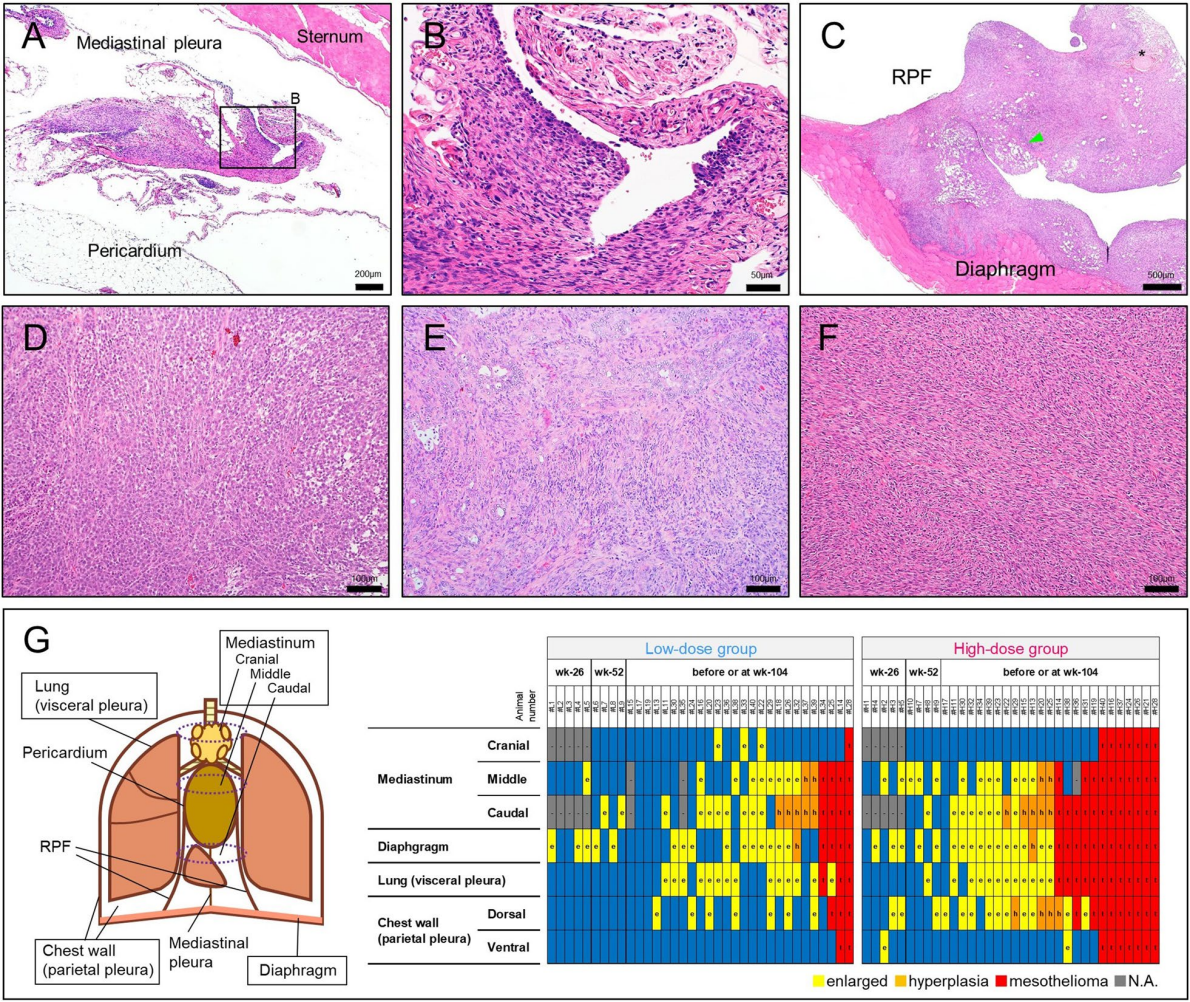
Malignant pleural mesotheliomas found in MWNT-7-treated rats. **A** Early stage of mesothelioma in a rat of the high-dose group at week 104. The partially thickened area with high cellularity is seen in the mediastinal pleura. **B** Higher magnification of (**A**). Growth of mesothelial cells is distinguished from a simple fibrotic lesion (upper half of the photograph). Surface elliptical or polygonal cells appear to be transitioning to fusiform cells. **C** Later stage of mesothelioma in a rat of the high-dose group sacrificed at week 81. A thick mass covers the RPF and diaphragm. Tumor invasion is apparent in the diaphragm muscle, and the normal components of the RPF are buried in the tumor mass. Arrowhead, fat; asterisk, nerve fascicle. **D** Epithelioid mesothelioma in the pericardium of a rat sacrificed at week 93 (high-dose group). **E** Biphasic mesothelioma in the diaphragm of a rat sacrificed at week 81 (high-dose group). The acinar-like structures are mixed with a sheet of spindle cells. **F** Sarcomatoid mesothelioma in an RPF in a rat sacrificed at week 93 (high-dose group). **G** Summary of the pleural lesions in all the MWNT-7-treated rats. A schematic view of the pleural structure in the rat is shown on left side. Detailed anatomical structure of the mediastinum is shown in Additional File. 2: Fig. S1. On the right side, individual histological data with the type and location of the lesion are summarized according to the time points and progression of the pathogenesis

The incidence of malignant pleural mesotheliomas in the high-dose group was significantly increased compared with the control group (Table 3). Although the incidence of pleural mesothelioma in the low-dose group was without statistical significance, it is notable that 4 rats in the low-dose group developed pleural mesothelioma: one of these rats died at week 53 (Fig. 2B, arrowhead). At the terminal necropsy, most mesotheliomas were early-stage mesotheliomas; these mesotheliomas were recognized as small nodules or bumps comprised of elliptical or polygonal tumor cells and underlying connective tissue-like cells (Fig. 6A and B). In contrast, most late-stage mesotheliomas were found in moribund animals or animals that died before the end of the study period. In these rats, tumor cells were disseminated throughout the thoracic cavity, and formed large nodular or sheet-like masses, generally located in the mediastinum (Fig. 6C). All 3 histological types of mesothelioma, epithelioid, sarcomatoid, and biphasic, were found (Additional File. 1: Table S5). A detailed histological analysis of the entire pleural mesothelial tissues present in the MWNT-7-treated animals revealed that the mesothelioma was likely to occur at the caudal region of the mediastinum (Figs. 6G and Additional File. 2: Fig. S1). By contrast, in the cranial region, even if the mediastinal lymph nodes contained relatively larger amounts of MWCNTs (Fig. 4I), hyperplasias or early stage of mesotheliomas were not observed (Fig. 6G).

The incidences of other tumor types are summarized in Additional File. 1: Table S6. None of these tumors was MWNT-7-treatment-related. The incidence of the interstitial cell tumors was slightly lower in the high-dose group than in the control group, which is possibly due to the lower survival rate in the high-dose group (Table 1).

### Cytological and biochemical analyses of bronchoalveolar lavage fluid (BALF)

**Fig. 7:**
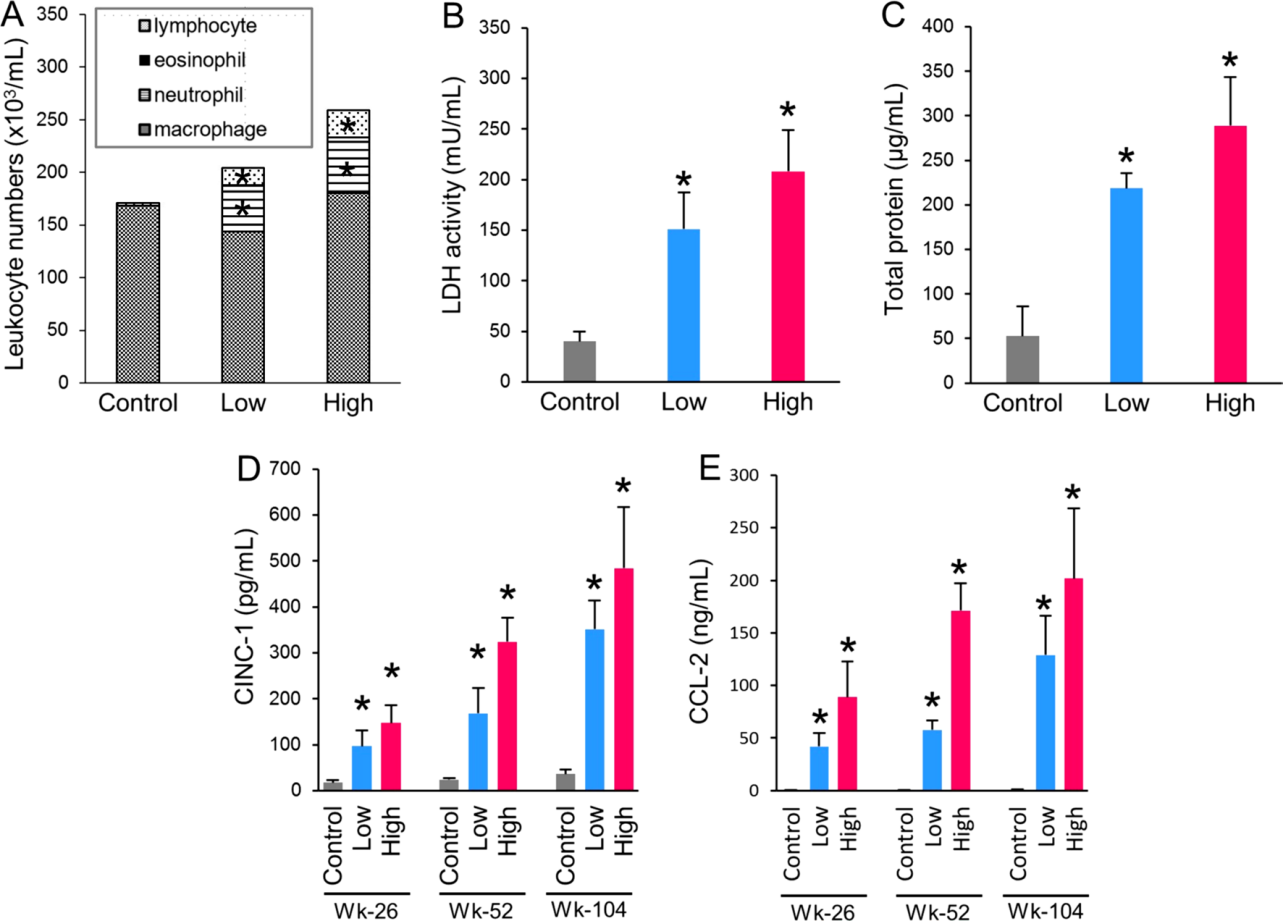
Cytological and biochemical analyses of the bronchoalveolar lavage fluid (BALF). **A** Cytological analysis at week 104. Total cells, lymphocytes, eosinophils, neutrophils, and macrophages per 1 mL of the BALF. **B** LDH activity at week 104. **C** Total protein concentration at week 104. **D** and **E** Concentration of CINC-1 (**D**), and CCL-2 (**E**). Significant increases in the MWNT-7-treated groups were found at all time points. Asterisks show significant differences (compared with corresponding control groups). Error bars show standard deviations

The number of neutrophils and lymphocytes in the BALF was significantly elevated in both MWNT-7-treated groups at week 104 (Fig. 7A). Lactate dehydrogenase (LDH) activity and total protein concentration in the BALF were significantly elevated in both MWNT-7-treated groups at week 104 (Fig. 7B and C). Two chemokines, cytokine-induced neutrophil chemoattractant-1 (CINC-1) and C–C motif chemokine-2 (CCL-2), were increased in both treatment groups in a dose- and time-dependent manner (Fig. 7D and 7E).

### Quantification and structural characterization of MWCNTs in the lung and pleural lavage fluid (PLF)

**Fig. 8:**
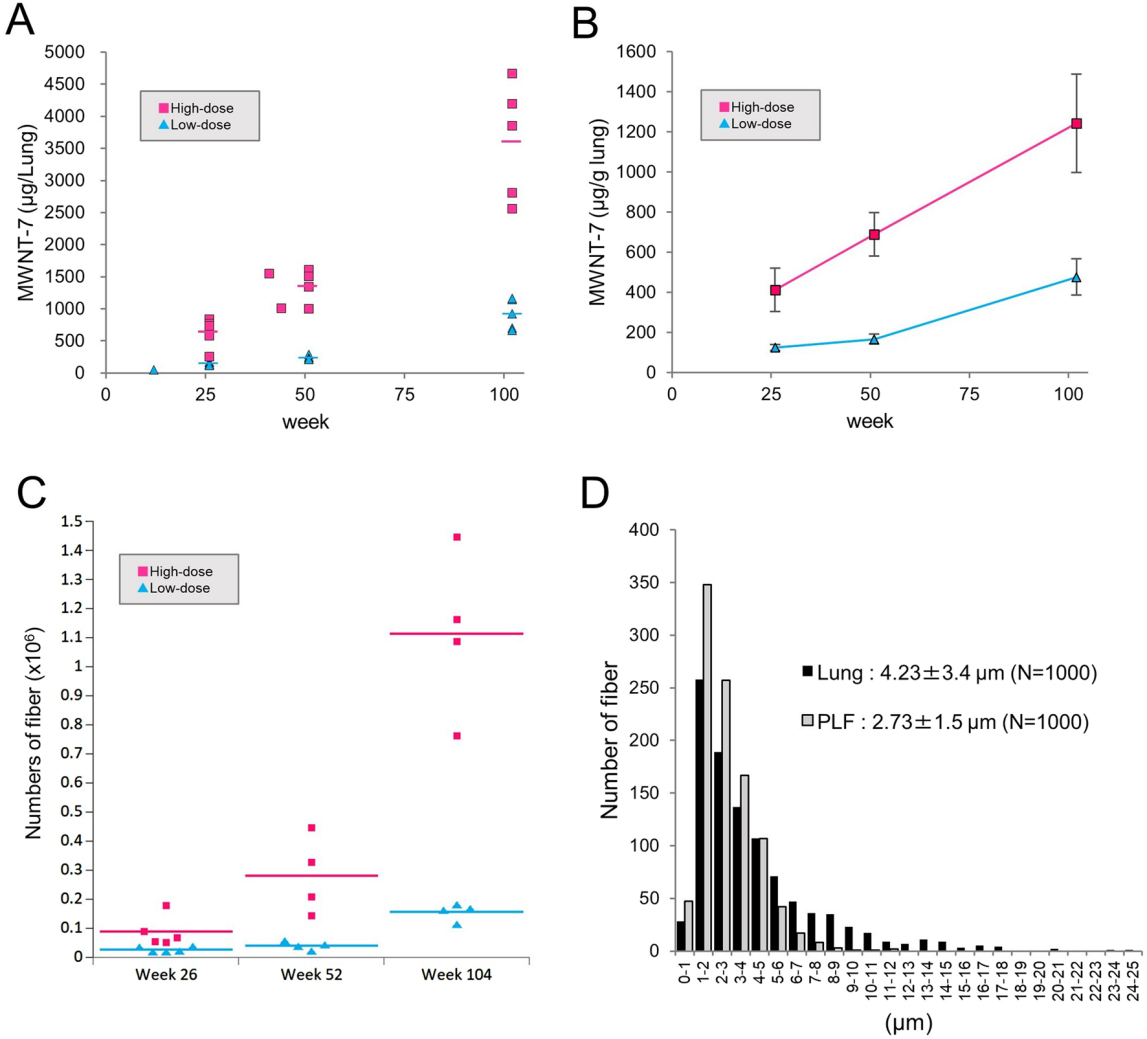
Quantifications and morphological characterization of MWNT-7 fibers in the lung and PLF. **A** Lung burden. Scatter plots for MWCNT weight per lung, including unscheduled samples (1 in the low-dose group and 2 in the high-dose group). Bars show mean values at 3 time points (weeks 26, 52, and 104). **B** Relative lung burden. Mean values of the MWCNT weight per gram lung at 3 time points are shown. Error bars show standard deviations. **C** Numbers of MWCNT fibers in the PLF at the 3 time points. Bars show mean values. **D** Histogram of the length distribution of MWCNT fibers in the lung (black bars) and PLF (gray bars)

Figure 8A shows the amounts of MWCNT in the lungs of animals sacrificed at weeks 26, 52 and 104 and in the lungs of 3 animals necropsied before the scheduled sacrifices. The lung burdens of both treatment groups was time dependent, and at week 104, the mean lung burden was 0.9 and 3.6 mg/lung in the low- and high-dose groups (Fig. 8A and Additional File. 1: Table S7). The ratio of the lung burden in the low versus high dose groups (0.9 versus 3.6 mg/lung) agrees with the administration dosage of 0.125 and 0.5 mg/kg body weight. The cumulative administered MWNT-7 doses were 1.2 and 4.8 mg/lung (Additional File. 1: Table S8). Approximately 50%–75% of the instilled MWNT-7 was found to have accumulated in the lung at each of the 3 time points. The amount of MWNT-7 per gram lung weights is also shown (Fig. 8B and Additional File. 1: Table S7).

The number of MWCNT fibers in the PLF was measured by SEM examination at the 3 time points (Fig. 8C), and the results again were dependent on time and dose. At week 104, there were 1.56 × 10^5^ and 1.11 × 10^6^ fibers in the low- and high-dose groups (Additional File. 1: Table S7).

The length distribution of MWCNT fibers obtained from lung tissue and the PLF is shown in Fig. 8D. The mean length of the fibers in the PLF was significantly shorter than in the lung (Table 4). For both samples, almost all fibers were single straight fibers. Notably, in the PLF sample, the percent of straight fibers was significantly higher than in the lung tissue and the percent of aggregated fibers was significantly lower than in the lung tissue.

**Table 4:** Comparison of the morphological MWNT-7 features between the lung and PLF

	Lung	PLF
Length		
Number of fiber examined	1000	1000
Average length (µm)	4.23 ± 3.4	2.73 ± 1.5*
Structural types		
Number of fiber examined	3823	1109
Straight (%)	3696 (96.66)	1090 (98.29*)
Aggregated (%)	121 (3.16)	18 (1.62*)
Branched (%)	6 (0.16)	1 (0.09)

Valules show the number of the fibers or length (Mean ± SD)

Values in parentheses are the percentage of each type of structure; 100 × number of each type of fiber ÷ number of fiber examined

*Significantly different from Lung (length, Student's t-test; rate of the structural types, Fischer's exact test)

## Discussion

The carcinogenicity of MWNT-7 in the rat lung was previously shown by Kasai et al*.* [11]. In the Kasai et al. study, 3 groups of rats were exposed MWNT-7 by whole-body inhalation at 0.02, 0.2, and 2 mg/m^3^ of MWNT-7 aerosol for 6 h/day, 5 days/week, for 104 weeks. The average lung burden in male rats at the end of the study period was 0.01, 0.15, and 1.8 mg/lung. The incidence of adenocarcinoma and combined adenoma and adenocarcinoma was significantly increased in the 0.2 and 2 mg/m^3^ groups. In contrast, in previous 2-year intratracheal instillation studies, the lung carcinogenicity of MWNT-7 was not higher than controls. For instance, when a total of 1.5 mg of MWNT-7 was instilled in rats, no lung tumors were reported, possibly due to the early death of the rats due to mesothelioma [21], In another 2-year study, in which MWNT-7 was used as reference material, instillation of 0.5 mg of MWNT-7 did result in 43% of the rats developing lung tumors at 2 years, however, the tumor incidence was not significantly increased compared with the control, possibly due to an abnormally high incidence of tumors in the untreated control group coupled with low animal numbers [28]. Therefore, the present study is the first to show overt lung carcinogenicity of MWNT-7 when administered by intratracheal instillation.

Intratracheal instillation studies can evaluate a large number of MWCNTs for the presence or absence of toxic/carcinogenic potential. In the present study, cumulative doses were set at more than 1 mg/lung, which is comparable with the 2-year inhalation study by Kasai et al. [11]. In our study, we found that rats that had accumulated approximately 3.6 mg MWCNT fibers in their lungs had a significantly increased incidence of lung and pleural tumors. Thus, administration of MWCNTs at doses that are comparable with the amount of fibers that accumulate in the lungs of rats exposed for 2 years to 2 mg/m^3^ MWNT-7 by whole body inhalation is reasonable for identifying MWCNTs that should be assessed further for hazard and risk characterization.

The primary reason for administering a higher dose in the high-dose group (accumulation of 3.6 mg/lung at the study termination) compared with the inhalation study (accumulation of 1.8 mg/lung in the 2 mg/m^3^ group at the study termination) was to examine whether the high lung burden could induce pleural mesotheliomas: in the inhalation study, rats did not develop pleural mesotheliomas. In our study, rats in the high-dose group did develop pleural mesotheliomas. Importantly, a large number of lung tumors were found at week 104. Although the tumor incidence in the high-dose group (39.3%; 11 of 28 animals) appears comparable with the report of Kasai et al*.* (32.0%; 16 of 50 animals in the 2 mg/m^3^ group), the early death of rats due to mesothelioma development likely precluded the lung tumor development. Thus, when calculating the incidence of lung tumors using only animals that survived to week 104, the incidence reaches a level of 75% (9 of 12 animals) in the high-dose group.

The mechanism of fiber-induced carcinogenesis is thought to be chronic inflammation, oxidative stress, and tissue injury and repair, leading DNA damages and mutation [12, 29, 30]. Our histological, biochemical, and cytological data demonstrated that macrophage-related inflammation persisted from the initial 26 week sacrifice to the end of the experimental period and that the level of inflammation increased during the experimental period. Considering the evidence that macrophages produce reactive oxygen species (ROS) during the phagocytic response to MWCNT exposure [62, 63], such persistent and increasingly elevated responses likely resulted in the high incidence of atypical hyperplasias and tumors in the lung at week 104. Recent studies of in vitro long-term exposure models and examining leukocytes from MWCNT-exposed workers suggest that MWCNT exposure can affect telomere length [31, 32]. Epigenetic alterations induced by MWCNTs have been suggested to occur [33]. It is possible that the difference of latency in the induction of rat neoplasms in the lung epithelium (2 years) and pleural mesothelium (less than 1 year) found in the present study may be accounted for by these complex mechanisms. Overall, the carcinogenic effects of long persistent fibers in the tissue requires further investigation.

**Fig. 9:**
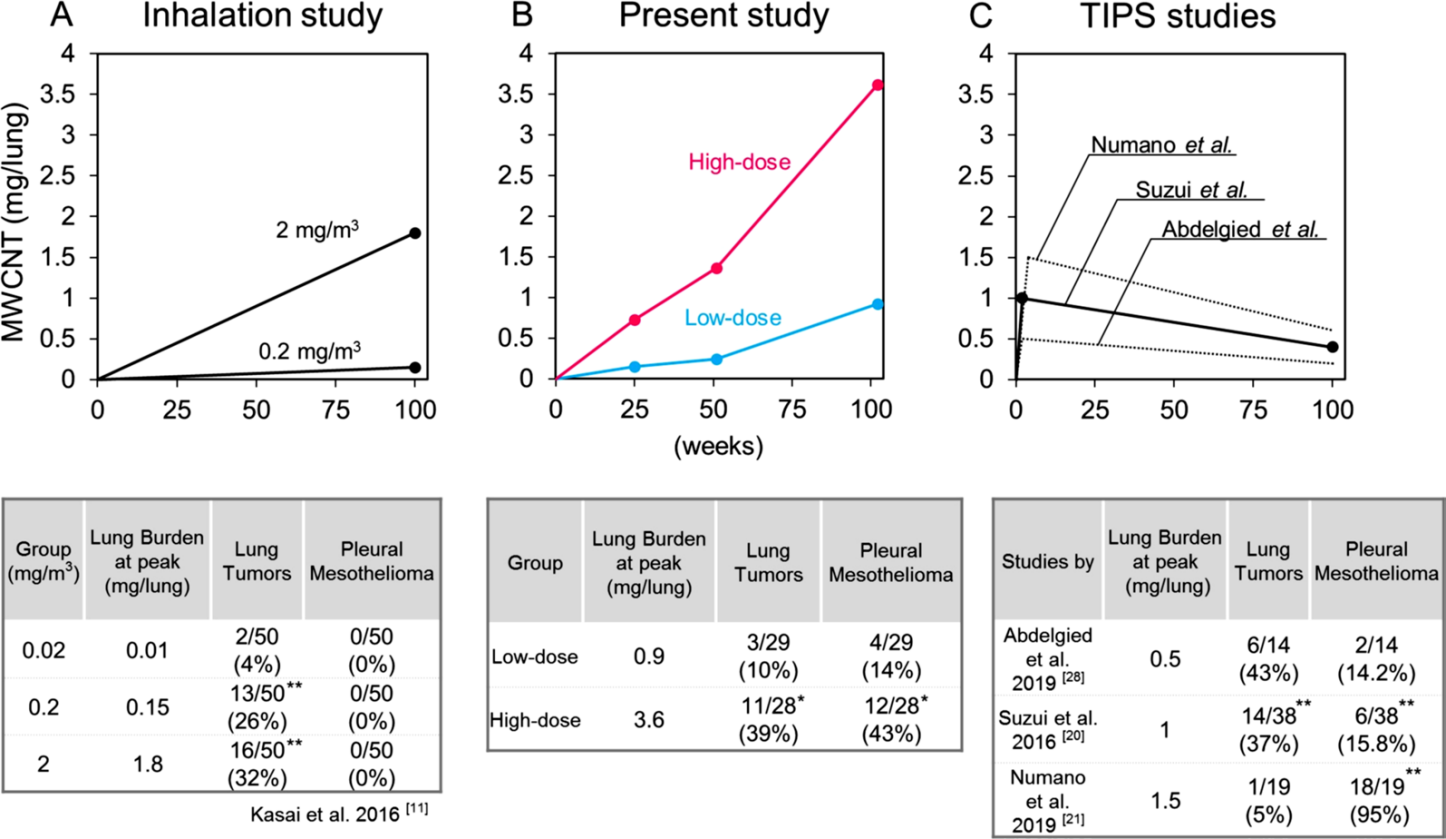
Comparisons of the AUC shape of MWCNT lung burden and tumor incidences. Upper: AUC curves of the lung burden of MWNT-7 during the experimental period in 3 types of experiments: inhalation study (results of only male rats were shown here) [11] (**A**), present study (**B**), and TIPS studies [20, 21, 28] (**C**). Circles show data points of the lung burden. In the studies by Abdelgied et al. and Numano et al., the lung burden was not measured; thus, predictive graphs are described by dotted lines, based on the dosages and data of Suzui et al. [20]. All lung burdens were measured by the same method (by Ohnishi et al.[60, 61]). Lower: summaries of peak lung burdens and incidences of lung tumor and pleural mesothelioma in each experiment. *, **: significant differences in each study. Test materials were MWNT-7 in all experiments except for a study by Suzui et al*.* (MWCNT-N) [20]

An area under the curve (AUC) of the burden of a test material is generally critical in the evaluation of a chronic effect. In the present study, as in inhalation tests of insoluble particulates, the amount of materials deposited in the lung gradually increased as the experiment proceeded because some fraction of the inhaled materials remained in the lung after each exposure period. Thus, the AUC is shaped like a right triangle during the 2-year experimental period (Fig. 9A). This differs from inhalation of gaseous material which doesn’t accumulate and results a constant burden level, giving a rectangular AUC. Our measurement of lung burden showed that the AUC was similar to the inhalation study, *i.e.*, an “increasing type” triangle (Fig. 9B). In contrast, the experimental design of previous intratracheal instillation studies using TIPS consisted of a short administration phase at the beginning of the experimental period followed by a subsequent 2-year non-treatment phase. Thus, in this type of study, the lung burden peaked at the end of the administration phase and decreased gradually to the end of experiment, resulting in a “decreasing type” triangle or trapezoid AUC (Fig. 9C). In two studies that used a lower fiber burden than our present study—Suzui et al*.* administered 1 mg MWCNT-N to rats and Abdelgied et al*.* administered 0.5 mg MWNT-7 to rats [20, 28]—although the peak lung burden levels of these studies (1 mg/lung and 0.5 mg/lung at week 2) were similar to or lower than that of the low-dose group in the present study (0.9 mg/lung at week 104), these 2 studies found higher incidences of lung tumors than our study (Fig. 9B and C). This suggests that an experimental design with a “decreasing type” of AUC is likely to lead to a higher tumor incidence than an experimental design with an “increasing type” of AUC. Most likely, this is because a “decreasing type” of AUC protocol provides the highest level of exposure to the test material at the beginning of the experiment while an “increasing type” of AUC protocol provides the highest level of exposure to the test material at the end of the experimental period. Thus, the protocol used will have a pronounced effect on the amount and duration of exposure of the test material [23].

The postulation that the TIPS-type exposure is more likely to induce tumors is especially applicable to pleural mesothelioma. In a previous study by Numano et al*.* using TIPS protocol, the peak value of the MWNT-7 lung burden (1.5 mg/lung) was lower than that of the high-dose group in the present study (3.6 mg/lung) [21]. However, their study resulted in a much higher incidence of mesothelioma (95%; Fig. 9C). For the development of pleural mesothelioma, translocation of a sufficient amount of fibers into the pleural cavity must occur, and this likely requires a long period of time and/or a high amount of fibers in the lung. It needs to be noted that once a substantial amount of MWCNT fibers attack the mesothelial tissue in rats, mesothelioma emerges within a short period of time. When carcinogenic MWCNTs were intraperitoneally injected into rats, peritoneal mesothelioma arose within 6 months after a single injection, and the mesothelioma caused deaths within 12 months [1, 34–36]. Similarly, in the study by Numano et al*.*, the first pleural mesothelioma was found about 7 months after the end of the instillation phase, and most of the animals died from malignant pleural mesotheliomas before the end of the study period. Interestingly, in the present study, the lung burden of the high-dose group reached approximately 1.5 mg/lung at week 52 (equal to the peak value reported by Numano et al*.*), and 6 months after that, a series of mesothelioma-related deaths occurred. By contrast, mesotheliomas were sporadically observed in the low-dose group. Thus, repetitive intratracheal administrations may enable the MWCNT fibers to efficiently attack the pleural mesothelium and induce mesothelioma, and importantly, delayed induction of mesothelioma by repetitive intratracheal administrations also allows the pulmonary toxicity of MWCNTs to manifest before the rats dies from mesothelioma. Notably, in inhalation studies, if the total dosage exceeds a potential threshold (*e.g.*, 1.5 mg/lung or so) and a sufficient time remains until the end of the experiment, pleural mesotheliomas will be induced. However, for a short lived experimental animal such as a rat, this may require exposure to an extremely high level of fibers (compare Fig. 9A, B). Overall, an instillation protocol with a decreasing AUC shape may be preferable for testing induction of mesotheliomas in addition to other fiber-induced toxicities.

Translocation pathway of the MWCNT fibers from the lung parenchyma to the pleural cavity is largely elusive. According to Morrow [37], a large fraction of insoluble particles deposited in the lung are cleared by the pulmonary lymphatic drainage toward a hilar region (so-called deep set drainage) and eventually conveyed to a systemic lymphatic flow. Indeed, many MWCNT fibers were evident in the BALT and mediastinal lymph nodes in previous studies and in our study [8, 20–22, 38]. As shown previously, MWCNTs administered via the airway were distributed in various organs such as the liver, kidney, and brain [11, 39]. These fibers appeared to be translocated via the systemic circulation of lymph and/or blood, and the pleural cavity may be one of the destinations. On the other hand, insoluble materials deposited in the parenchyma are also thought to be eliminated via the pleural lymphatics (so-called pleural drainage) albeit the contribution of the drainage is not high [37]. Donaldson et al*.* proposed that MWCNT fibers penetrate into the pleural cavity via the visceral pleura and translocate to the parietal pleura via the lymphatic flow [40, 41]. The route through the visceral pleura has been addressed in several in vivo studies. Mercer et al*.* showed rapid distribution of MWNT-7 fibers on the visceral pleura after instillation by pharyngeal aspiration, and presented a clear image of fibers penetrating the visceral pleura using field emission scanning electron microscopy [6]. Similarly, Xu et al*.* revealed that a long and rigid fiber (MWCNT-L; 8 µm in length and 150 nm in width) instilled by intratracheal administration could pierce the visceral pleura and penetrate the parietal mesothelium in rats [42]. Our microscopic observation also revealed an abundance of MWNT-7 fibers piercing the visceral pleura. Miserocchi et al*.* proposed that translocation of inhaled asbestos fibers to the pleural cavity is usually achieved by the systemic lymphatic route, but direct movement into the pleural cavity through the visceral pleura is also possible when the pulmonary interstitial pressure is increased by inflammatory and edematous change [43, 44]. According to fluid dynamic models, movement of MWCNT fibers across the visceral pleura would occur as a result of the inflammatory condition of the visceral pleural interstitium that was histologically demonstrated in our study.

Although which route of translocation of the fibers is more critical for mesotheliomagenesis is still obscure, Miserocchi et al*.* also suggested that thin and short asbestos fibers can travel longer distances due to a lower steric hindrance [44]. In a high-resolution electron microscopic analysis, 90% of the asbestos fibers in the tissue samples of human malignant pleural mesothelioma were short and ultrathin (< 5 µm in length and < 250 nm in width), and the fibers in the extrapulmonary samples (pleural plaque and mesothelioma) were smaller than the fibers from the lung [45]. Thus, the authors concluded that short, thin asbestos fibers were likely to contribute to the induction of mesothelioma. In the present study, the fibers in the PLF were significantly shorter than those in the lung. MWCNTs are also mostly thin, which is comparable with the ultrathin fibers of asbestos. In addition, a series of data from TIPS studies have an interesting implication. While thick and long fibers such as MWNT-7 (80–90 nm in width) and MWCNT-N (60 nm in width) resulted in high incidences of pleural mesothelioma [20, 21], a much thicker carbon nanotube, MWCNT-A (150 nm in width) did not induce mesothelioma [22]. Because a thick and long MWCNT, SD-1 (177 nm in width, similar to MWCNT-A) was found to be highly carcinogenic in a rat peritoneal injection model [34], the lack of induction of pleural mesothelioma in the TIPS experiment may be attributed to failure of translocation into the pleural cavity. Furthermore, Xu et al. revealed that a much thinner fiber, MWCNT-S (15 nm in width), forming a tangled structure, did not translocate to the pleural cavity [42]. In the present study, the straight fibers were observed as the predominant structure in the PLF. Taken together, the straight, rigid, and relatively shorter fibers may tend to translocate to the pleural cavity, and thus, contribute to mesotheliomagensis in the rat pleura, which differs slightly from the results of the peritoneal injection model and the Stanton hypothesis [34, 46, 47].

The most important difference in the results between the intratracheal instillation test and inhalation test is the induction of the pleural mesothelioma. We have speculated that the pleural burdens would be higher in the intratracheal instillation or pharyngeal aspiration studies than in the inhalation study. However, there was little information about the quantification of MWCNT fibers in the pleural cavity in long-term experiments. Herein, our SEM observations revealed that the number of MWCNTs in the PLF increased with time and dose, in line with the results of the lung burden. Importantly, the numbers of fibers in the PLF were approximately 2-orders of magnitude greater than that reported by Kasai et al*.*: 1,468 fibers were found in the PLF of a male rat in the 2 mg/m^3^ group at week 104 [11]. According to their calculation, this value corresponds to only 0.0000091% of the lung burden. Mercer et al*.* quantified extrapulmonary MWCNTs in C57BL mice after repeated exposures at 5 mg/m^3^ for 12 days by a whole-body system. They found a lung burden of 28.1 µg/lung and that 23.7 ± 7.6 fibers were in the PLF (corresponded to 0.0000018% of the lung burden) at 336 days after the exposure period [39]. The present study showed a much higher proportion of fibers in the PLF compared to the inhalation studies: the number of fibers in the PLF corresponds to 0.00093% and 0.00169% of the lung burden in the low- and high-dose groups based on a conversion of the weight of MWCNT into the fiber numbers (1 g = 1.83 × 10^13^ fibers). This extremely large difference in the number of translocated fibers is very likely associated with the difference in the induction of mesothelioma in the present study and study by Kasai et al*.*. Even in the low-dose group (0.9 mg/lung at week 104), the number of fibers in the PLF was greater than that of the 2 mg/m^3^ group in the inhalation study. Although the incidence of mesothelioma in the low-dose group did not show a statistically significant increase, in light of the very low occurrence of spontaneous pleural mesotheliomas in F344 rats (0.03%) [48], the induction of pleural mesotheliomas in 4 animals (13.8%) is very likely to be treatment related. This result sharply contrasts with that of Kasai et al*.* in which none of the 50 male or 50 female rats exposed to 2 mg/m^3^ MWNT-7, resulting in lung burdens of 1.8 and 1.2 mg/lung, respectively, developed pleural mesotheliomas. One of the possible factors accounting for this difference of amount of fibers in the PLF is employing Taquann treated MWNT-7 which resulted in a high amount of single fibers (98.8%) compared with the bulk MWNT-7 (92.0%; Additional File. 2: Fig S3) under our sample dispersing method. However, the method of aerosolization used by Kasai et al*.* also resulted in most of the fibers in the inhalation being single fibers [11]. On the other hand, administration by TIPS of sonicated bulk MWNT-7 was shown cause high incidence of pleural mesothelioma in rats [21]. Thus, the pretreatment by Taquann is unlikely to be linked to the large difference in the numbers of MWCNT fibers in the PLF of the present study and the inhalation study. Another possible difference is the length of the fibers. The average length of the fibers found in the lungs of the rats in the inhalation study was 5.8–5.9 µm and the average length of the fibers found in the lungs of the rats in our study was 4.23 µm. While this difference in length may have affected translocation into the pleural cavity, it is unlikely to have caused the almost 2-3 orders of difference in the number of fibers found in the PLF. Therefore, the different methods of exposure to MWNT-7,* i.e*., intratracheal instillation versus inhalation, is possibly more critical for the inducibility of pleural mesothelioma than other factors such as the total lung burden, AUC shape, or pretreatment of MWNT-7.

Because intratracheal administration delivers the test material as a bolus, the dose rate is much higher than that in inhalation studies. Additionally, drainage of the liquid in which the MWNT-7 was dispersed from the lung may occur just after instillation. Both of these factors may possibly enhance the translocation of the fibers into the pleural cavity (maybe via “pleural drainage” or across the visceral pleura) in intratracheal instillation studies. Taking into consideration the facts that the inhalation study also showed a dose-dependent induction of mesothelial hyperplasia and that the translocation across the visceral pleura is thought to be one of the plausible routes of asbestos translocation in the pathogenesis of human malignant pleural mesothelioma, we believe that the intratracheal instillation method is an excellent alternative to inhalation for identifying and ranking the hazard of fibrous materials.

The location where MWCNT fibers that accumulate in the pleural cavity and the primary site of mesothelioma suggest by analogy to human asbestos-related conditions, *i.e.,* black spots, pleural plaque, and mesothelioma [40], that the parietal pleura is the likely region where fiber induced pleural mesothelioma develops in rats. However, Suzui et al. after TIPS administration of MWCNT-N found that malignant pleural mesotheliomas tended to develop at the mediastinum, rather than at the chest wall [20]. In addition, small nodules of the mesothelioma (mesothelioma at an early phase in appearance) were found on the surface of the pericardium and the RPF in a rat treated with potassium octatitanate fibers [28]. Similarly, our detailed histological analysis demonstrated that a probable primary site of mesothelioma was the mediastinum, especially, the caudal region including the mediastinal pleura and the RPF. Like humans, rodents have stomata in the chest wall [49–51]; however, they have another stomata-enriched structure, the RPFs, that extend from the pericardium to the sternum and diaphragm as depicted schematically in Additional File. 2: Figs. S1C and S1D [25, 26]. In humans, the RPFs are absent and the pericardium is connected to the diaphragm by the mediastinal pleura. The RPF is a major lymphatic drainage site in dogs, rabbits, rats, and mice when particles were administered by intrapleural injection [51–53]. In addition, the RPF contains numerous lymphatic-associated tissues and serosa-associated lymphatic clusters (SALCs), which are thought to play central roles in the immune response in the pleural cavity [26]. Lymphocytes and macrophages accumulate in the SALCs of the RPF and macrophages and lymphocyte-rich SALCs are present in the mediastinum. The development of mesothelioma induced by fibrous materials is associated with chronic inflammation mainly by fiber-phagocyting cells and immunosuppressive conditions such as functional impairment of natural killer cells and cytotoxic T lymphocytes, most prominently by regulatory T cells and myeloid derived suppressive cells [54–58]. In the present study, inflammatory or hyperplastic lesions were frequently found around the RPF, and the lesions harbored singlet MWCNT fibers and numerous leukocytes such as macrophages/monocytes, lymphocyte, and eosinophils. Collectively, this suggests that accumulation of both fibers and immune cells probably render the caudal mediastinum susceptible to mesotheliomagenesis.

## Conclusions

Repeated administration of MWNT-7 to male F344 rats by intratracheal instillation over the course of 2 years resulted in dose-dependent induction of lung tumors and pleural mesothelioma. This is the first report demonstrating that MWNT-7 induces both lung tumors and pleural mesotheliomas after administration to a single group of rats. In this study, the lung burden of MWCNT gradually increased over the experimental period, similarly to a 2-year inhalation study. This is in contrast to the previous studies using TIPS in which lung burden peaked at the beginning of the experiment. A comparison between experiments administering approximately 1 mg/lung of MWNT-7 shows that the tumor incidences in the present study (low-dose group) tended to be lower than the tumor incidences in the experiments that administered MWNT-7 by TIPS. This suggests that the AUC shape may affect the outcomes of carcinogenicity tests.

Whether pleural mesothelioma is induced appears to be linked to the exposure methods, with intratracheal instillation, but not inhalation exposure, inducing mesothelioma. We demonstrated that the mediastinum, especially in the caudal region, was the possible primary site of MWCNT-induced pleural mesothelioma development in rats. Although the path through which the fiber translocates from the lung to the pleural cavity is still unclear, we showed that a substantial amount of fibers were present in the pleural cavity at 26, 52, and 104 week.

## Methods

### Preparation of the test material

The MWCNT, MWNT-7 (also called as Mitsui-7, XRNI-7, or MWCNT-7; lot, 060 125-01 k), was obtained from Mitsui Chemicals (Tokyo, Japan) and was fully characterized in our previous reports [24, 34]. Since intratracheal instillation bypasses the upper respiratory tract, it may deliver fiber aggregates, which are presumed to be entrapped in the nasal cavity in inhalation tests, to the lung parenchyma. Thus, the bulk materials were pretreated with a filtration method, the Taquann method, which removes the agglomerates and aggregates of fibers without changing the size distribution of the fibers by employing a fine filtration (a 53-µm mesh) and a critical point drying technique [24] (Fig. 1 and Additional File. 2: Fig. S3). Thereafter, the Taquann treated-MWCNT was baked at 200 °C for 2 h in a dry heat sterilizer for the elimination of endotoxin. The MWCNT was suspended in saline containing 0.1% Tween 80 at a concentration of 0.125 (low-dose group) and 0.5 mg/mL (high-dose group) and then sonicated for 30 min using an ultrasonic bath (100 W; US-702, SND, Nagano, Japan). To minimize aggregation, the suspension was again sonicated in the animal room immediately before the administration.

### MWCNT characterization

The suspension used for administration was diluted at a concentration of 0.01 mg/mL with pure water containing 0.1% Triton X-100, and 1 µL of the diluted suspension was placed on an inorganic aluminum oxide membrane filter (Whatman® Anodisc, Cytiva, Marlborough, MA, USA). The filter was coated with gold and viewed by SEM (Quanta™ FEG250; Thermo Fisher Scientific, Waltham, MA, USA) at 10–20 kV. A total of 1000 fibers were randomly photographed at magnifications of 5,000 and 10,000 for length measurements and 60,000 for width measurements. The morphological classification and length and width measurements of the MWCNT fibers were performed by ImageJ software (NIH, Bethesda, MD, USA).

The secondary diameter in the suspension containing MWCNT at 0.01 mg/mL was analyzed by DLS (Zetasizer Nano, Malvern, Worcestershire, UK).

### Animals and treatment

A total of 150 of 5-week-old male specific pathogen-free Fischer 344 (F344/DuCrlCrlj) rats were purchased from Jackson Laboratories Japan (Kanagawa, Japan). Male rats were chosen because of their susceptivity to the development of lung tumors [11] and for comparisons with TIPS studies [20–22]. The rats were housed in a polycarbonate cage (3 rats per cage) in a room maintained at a temperature of 23 ± 0.1 °C and 53.1 ± 7.9% relative humidity on a 12 h light–dark photophase cycle, and given standard CE-2 basal diet (CLEA Japan, Tokyo, Japan) and drinking water via a bacterial filter ad libitum. After a 2-week quarantine and acclimation period, the animals were randomly divided into 3 groups: vehicle control group, low-dose group, and high-dose group, which consisted of 40, 55, and 55 animals, respectively (Additional File 1 Table S1).

Intratracheal administration of MWCNT to rats was performed 26 times at intervals of 4 weeks from 9 weeks of age, according to previous studies [18, 59]. Briefly, the rats were deeply anesthetized by inhalation of 3% isoflurane (Pfizer, New York, NY, USA), held on a holder inclined at 45°, and then the vehicle or the MWCNT suspension was instilled at 1 mL/kg body weight through the larynx into the lung using a feeding cannula (20-gage; Natsume Seisakusho, Tokyo, Japan) connected to a 1.0-mL syringe (Terumo, Tokyo, Japan). The animal was kept in the holder until its usual respiratory rhythm was recovered and then placed back in the housing cage. The MWCNT dosage, *i.e.*, 0 (control), 0.125 (low dose) or 0.5 (high dose) mg/kg body weight, was decided based on a pilot study to examine the time course of the lung burden.

General conditions were observed twice daily, and body weight was measured every week.

Satellite animals were sacrificed at week 26 and week 52 after the first administration to monitor histological features and lung burden levels. At each time point, 5, 10, and 10 animals were sacrificed for the control, low, and high-dose groups, respectively. For each group, 30 animals were included for carcinogenic assessment (Additional File. 1: Table S1).

### Autopsy and sample collection

At the interim sacrifices (weeks 26 and 52) and termination of the study (week 104), animals were killed by exsanguination through the abdominal aorta under 3% isoflurane anesthesia, and macroscopically examined. Animals that died or were humanely euthanized before the end of the experiments were similarly treated.

The number of animals used in each analysis is summarized in Additional File. 1: Table S1. BALFs were collected from 5 animals from each group in the interim sacrifices and 10 animals from each group at the terminal necropsy. To lavage only the right lung, the left bronchus was tied with a thread, and the right lung was lavaged 2 times with 4 mL of phosphate-buffered saline at a water pressure of 30 cm. The washout was centrifuged at 402 g at 4 °C for 10 min. The supernatant was collected for biochemical analyses, and the pellet was gently resuspended for cytological analysis. For measurement of the lung burden, a whole lung was obtained from 4 or 5 animals from both the MWCNT-treated groups at the interim and terminal necropsies. PLFs were collected from the animals used for measuring the lung burden at the interim and from 15 animals of the terminal necropsies. The thorax of the rat was lavaged once with 8 mL of saline using a syringe with a needle, and the washout was collected for SEM analyses.

At the terminal necropsy, major organs, trachea, lungs, parietal pleura (diaphragm and chest wall), heart, spleen, bone marrow, mediastinal lymph nodes, thymus, tongue, salivary glands, esophagus, stomach, small intestine, large intestine, liver, pancreas, kidneys, urinary bladder, adrenal glands, pituitary gland, thyroid glands with parathyroids, testes, epididymis, seminal vesicles, prostate, mammary glands, muscle, bone, brain, spinal cord, eyes, Harderian glands, Zymbal’s glands, and skin, were collected from all animals for histopathological examination. The brain, heart, lungs, liver, spleen, kidneys, adrenal glands, and testes from 10 animals from each group were weighed.

### Hematological analysis

The blood samples obtained from 10 animals in each group at the terminal necropsy were analyzed with an automatic blood cell analyzer (KX-21NV, Sysmex, Hyogo, Japan). Differential counts of leukocytes were made by a light microscopic observation of smeared specimens stained following a routine May–Grunwald–Giemsa protocol.

### Histopathology

All dissected organs and tumor masses were fixed in 10% neutral buffered formalin, embedded in paraffin, sectioned (4 μm thickness), and stained with hematoxylin and eosin. All 5 separate lung lobes were evaluated. The entire mediastinum including the pericardium, heart, mediastinal pleura, and RPF were embedded in agarose, and then trimmed, following the routine procedure for paraffin sections. Among a series of samples along the cranial–caudal axis, 3 levels or more were histologically examined (Additional File. 2: Figs. S 1D and S1E).

The severity of non-neoplastic lesions was graded on a 5-point scale of no/minimal (0), slight (1), mild (2), moderate (3), or marked (4). Proliferative lesions of the lung and pleura were blindly examined by more than 4 pathologists, and the final diagnosis was made by Dai Nakae, one of the authors, who is a board-certified pathologist of the Japan Society of Toxicologic Pathology (Diplomate of JSTP) and the Japan Society of Pathology. Pleural mesotheliomas were classified into 3 types, epithelioid, sarcomatoid, and biphasic. Biphasic mesothelioma was diagnosed when both epithelioid and sarcomatoid components were > 10% of the tumor masses collected from the thoracic cavity.

For immunostaining, antigen retrieval was performed in Tris–EDTA buffer (pH 9.0) using an autoclave for 60 min or 10 mM citrate buffer (pH 6.0) using a microwave for 15 min, followed by the inactivation of endogenous peroxidase by immersion in H_2_O_2_. After blocking with Protein Block (X0909; Agilent technologies, Santa Clara, CA, USA) for 20 min at room temperature, the sections were treated with primary antibodies: thyroid transcription factor-1 (TTF-1, ab72876, abcam, Cambridge, UK), or mesothelin/c-ERC (28001, Immuno Biological Laboratories, Gunma, Japan) for 1 h at room temperature. Diaminobenzidine signals were detected with a horseradish peroxidase-secondary antibody conjugate (K4061, Agilent Technologies) according to the manufacturer’s instructions.

To count the MWCNT fibers penetrating the visceral pleura, a new region of the knife blade was used to cut each section block, with a unidirectional cut from outside of the tissue toward the center of the parenchyma, to minimize the possibility that the fibers were artificially positioned on the visceral pleura. To enhance the contrast between the tissue and MWCNT fibers, the sections were stained with Kernechtrot solution.

### Cytological analysis in the BALF

Cells recovered after centrifugation of the BALF were stained with Turk’s solution, and the number of leukocytes was determined using a counting chamber slide. For differential leukocyte counts, cytoslides were prepared (CF-120, Sakura Finetek Japan, Tokyo, Japan), stained with May–Grunwald–Giemsa, and microscopically examined.

### Biochemical analyses in the BALF

LDH activity in the BALF was determined by an assay kit (Takara Bio, Shiga, Japan) and an LDH standard (Roche Diagnostics, Mannheim, Germany). Concentrations of total protein in the supernatant of the BALF were measured by a kit (Cytiva) following the manufacturer’s instructions. Levels of CINC-1 ( also known as KC, CXCL-1, and GRO-α) and CCL-2 in the BALF supernatant were individually analyzed using ELISA kits: CINC-1, RCN100 (R&D systems, Minneapolis, MN, USA); CCL-2,  ELR-MCP-1 (RayBiotech Life, Peachtree Corners, GA, USA).

### MWCNT measurement in the lung and PLF

MWCNT quantification in the lung was performed as previously described [60, 61]. Briefly, fixed whole lung samples were digested by a strong alkali solution (Clean 99, K200; Clean Chemical, Osaka, Japan), and after washing and removing organic debris with sulfuric acid, benzo[ghi]perylene (B(ghi)P) (CAS: 191–24-2, Sigma-Aldrich®, B9009, Merck, Kenilworth, NJ, USA) was added to the sample solution containing MWCNTs. The sample solution was immediately and thoroughly mixed by an ultrasonic homogenizer (UH-50, SMT, Tokyo, Japan) so that B(ghi)P was adsorbed to the MWCNT. The solution was then passed through a membrane filter (Whatman® Nuclepore, 111,109, Cytiva). The B(ghi)P that interacted with the MWCNT on the filter was desorbed into acetonitrile. Finally, the B(ghi)P was analyzed by an UHPLC system (Nexera X2, Shimadzu, Kyoto, Japan) with a reversed phase column (ACQUITY UPLC BEH C18, Waters, Milford, MA, USA).

Counting and morphological analyses of MWCNT fibers in the PLF were performed by SEM, as described above. The PLF was centrifuged at 20,000 g for 1 h, and the pellet was digested with Clean 99 for 2 h. After washing once with pure water containing 0.1% of Triton X-100, the precipitate was resuspended at a volume of 50 µL. Four or 5 animals were used to count the numbers of fibers at each time point. A total of 1,000 fibers randomly selected from these samples were further examined for length measurement and a morphological classification.

Similarly, a total of 1,000 fibers in the digested lung tissue, which was used in the lung burden measurement, were analyzed for the length distribution and morphology.

### Statistical analysis

Body weight, organ weight, hematology, cell counts in the BALF, and biochemical parameters in the BALF were analyzed by Dunnett’s multiple comparison test. Survival curves were plotted according to the Kaplan–Meier method, and the log-rank test was used to detect significant differences in survival rates between the treated groups and the vehicle control group. Histological scoring of non-neoplastic lesions was analyzed by Steel’s multiple comparison test. The difference in the fiber length between the lung and PLF was analyzed by Student’s t-test. Differences in the incidence of neoplastic or non-neoplastic lesions from the control group were analyzed by Fisher’s exact test. Two-tailed tests were used for all statistical analyses. Differences in values were deemed significant when *p*-values were less than 0.05. Statistical analyses were performed using StatLight software (Yukms, Kanagawa, Japan).

## Supplementary Information

**Additional file 1 **: Table S1. The disposition of the animals in this study. Table S2. Deaths prior to the termination among the animals used for the carcinogenicity test. Table S3. Major organs weights. Table S4. Hematological analyses for rats in the terminal necropsy. Table S5. Histological classification of all pleural mesothelioma cases found in this study. Table S6. Incidences of other tumors. Table S7. Amounts of MWCNT in the lung and PLF. Table S8. Mean doses for each administration.

**Additional file 2**: Fig. S1 Anatomical structure of the mediastinum and histological sample preparations of the pleura. Fig. S2. MWNT-7 fibers piercing the visceral pleura. Fig. S3. Characterization of the bulk MWNT-7: a comparison with Taquann-treated MWNT-7.

## Data Availability

All data generated or analyzed during this study are included in this published article.

## Abbreviations

AUC: Area under the curve
BALF: Bronchoalveolar lavage fluid
BALT: Bronchiolar associated lymphatic tissue
B(ghi)P: Benzo[ghi]perylene
CCL-2: C–C motif chemokine-2
CINC-1: Cytokine-induced neutrophil chemoattractant-1
LDH: Lactate dehydrogenase
MWCNT: Multiwalled carbon nanotube
PLF: Pleural lavage fluid
RPF: Retrocardiac pleural fold
SALC: Serosal-associated lymphatic cluster
SEM: Scanning electron microscope
TIPS: Intra-tracheal intra-pulmonary spraying
UHPLC: Ultra High Performance Liquid Chromatography
