# Supplementary material for: Two-year intermittent exposure of a multiwalled carbon nanotube by intratracheal instillation induces lung tumors and pleural mesotheliomas in F344 rats
Source: Part Fibre Toxicol. 2022 May 19;19:38. doi: 10.1186/s12989-022-00478-7 (PMC9118836; doi:10.1186/s12989-022-00478-7)
Supplement: Supplementary file 1 — Additional file 1 : Table S1. The disposition of the animals in this study. Table S2. Deaths prior to the termination among the animals used for the carcinogenicity test. Table S3. Major organs weights. Table S4. Hematological analyses for rats in the terminal necropsy. Table S5. Histological classification of all pleural mesothelioma cases found in this study. Table S6. Incidences of other tumors. Table S7. Amounts of MWCNT in the lung and PLF. Table S8. Mean doses for each administration. [file 12989_2022_478_MOESM1_ESM.pdf]

Table S1. The disposition of the animals in this study.

Time	herd		Control planned	Control actual	Low-dose planned	Low-dose actual	High-dose planned	High-dose actual	Item
Interim sacrifice at wk-26	Pathology		5	5	5	5	5	5	Histology, BALF
	Lung burden		-	-	5	5	5	5	Lung burden, PLF
Interim sacrifice at wk-52	Pathology		5	5	5	4[a)]	5	4[d)]	Histology, BALF
	Lung burden		-	-	5	4[b)]	5	4[e)]	Lung burden, PLF
Terminal necropsy at wk-104	Pathology (for the carcinogenicity assessment)	Before the termination	30	5	30	7[c)]	30	16[f)]	Histology
		Terminal		25		22		12	Histology (all)<br>BALF, PLF, organ weights, Hematology (10 animals each group)
	Lung burden	Terminal	-	-	5	5	5	5	Lung burden, PLF

a) One rat died during administration of the material (airway obstruction) at week 14. The lung sample was used for measuring MWCNT lung burden.

b) One rat died from unknown causes (week 60).

c) One rat died during an administration of the material (airway obstruction) at week 64.

d) One rat died during an administration of the material (airway obstruction) at week 40. The lung sample was used for measuring MWCNT lung burden.

e) One rat was cannibalized at week 44. The cause of death was undetermined. The lung sample was used for measuring MWCNT lung burden.

f) One rat died of a pituitary tumor at week 76 and conditions of his major organs were too bad to be examined. The other rat died during an administration of the material (airway obstruction) at week 76.

Table S2. Deaths prior to the termination among the animals used for the carcinogenicity test.

Group	Condition	Time of death (week)	Cause of death
Control	Sacrificed	61	Oligodendroglioma
	Sacrificed	69	Seminal vesicle adenoma
	Dead	76	Large granular lymphocytic leukemia (LGL)
	Sacrificed	92	LGL
	Sacrificed	93	Peritoneal mesothelioma
Low-dose	Sacrificed	38	Osteosarcoma (subcutis, hindleg)
	Dead	53	Pleural mesothelioma
	Sacrificed	66	LGL
	Sacrificed	66	Harderian gland adenocarcinoma
	Sacrificed	93	Pleural mesothelioma
	Sacrificed	95	Pituitary, pars distalis, adenoma
	Sacrificed	98	LGL
High-dose	Dead	63	LGL
	Sacrificed	75	Zymbal's gland carcinoma
	Dead	76	Bladder dysfunction
	Dead	79	Pituitary, pars distalis, adenoma
	Dead	80	Pleural mesothelioma
	Sacrificed	81	Pleural mesothelioma
	Sacrificed	81	Pleural mesothelioma
	Sacrificed	82	Pleural mesothelioma
	Sacrificed	85	LGL
	Dead	87	Pleural mesothelioma
	Sacrificed	89	LGL
	Sacrificed	89	Hemangiosarcoma (subcutis, neck)
	Sacrificed	93	Pleural mesothelioma
	Sacrificed	93	Pleural mesothelioma
	Sacrificed	98	Pituitary, pars distalis, adenoma
	Sacrificed	100	LGL and/or peritoneal mesothelioma

Table S3. Major organs weights

Group	Control	Low-dose	High-dose
Numbers of animals	10	10	10
Brain (g)	2.21 ± 0.04	2.22 ± 0.05	2.17 ± 0.06
Spleen(g)	1.10 ± 0.22	1.13 ± 0.3	1.04 ± 0.27
Liver (g)	13.3 ± 0.92	13.1 ± 1.3	12.9 ± 0.78
Kidneys (g)	2.71 ± 0.19	2.76 ± 0.2	2.66 ± 0.09
Adrenals (mg)	45.2 ± 12	48.5 ± 6.3	46.4 ± 8.1

Mean ± SD

Spleen: one rat bearing LGL in the low-dose group was excluded

Table S4. Hematological analyses for rats in the terminal necropsy.

Item		Control<br>N = 10	Low-dose<br>N = 9 1)	High-dose<br>N = 10
Total WBC	($x10^2/\mu L$)	13.50 ± 3.5	12.00 ± 2.8	13.60 ± 4.0
Neutrophil	($x10^2/\mu L$)	6.85 ± 1.9	5.23 ± 1.3	5.29 ± 1.7
Lymphocyte	($x10^2/\mu L$)	6.28 ± 2.1	6.23 ± 1.8	7.42 ± 2.4
Eosinophil	($x10^2/\mu L$)	0.10 ± 0.1	0.28 ± 0.2	0.54 ± 0.2*
Monocyte	($x10^2/\mu L$)	0.27 ± 0.2	0.26 ± 0.1	0.35 ± 0.3
RBC	($x10^4/\mu L$)	858.8 ± 130	902.9 ± 81	930.1 ± 140
HGB	(g/dL)	14.1 ± 2.2	14.8 ± 1.2	15.1 ± 1.9
HCT	(%)	45.9 ± 6.9	47.8 ± 3.9	49.0 ± 6.6
MCV	(fL)	53.4 ± 1.0	53.0 ± 1.0	52.8 ± 1.2
MCH	(pg)	16.4 ± 0.5	16.4 ± 0.5	16.3 ± 0.6
MCHC	(g/dL)	30.7 ± 0.7	30.9 ± 0.6	30.8 ± 0.4
PLT	($x10^4/\mu L$)	74.1 ± 18.1	69.0 ± 9.8	66.3 ± 8.7

Mean ± SD

1) One rat bearing LGL in the low-dose group was excluded

*Significantly different from Control (by Dunnett's test)

WBC, white blood cell count; RBC, red blood cell count; HGB, hemoglobin concentration; HCT, hematocrit level; MCV, mean corpuscular volume; MCH, mean corpuscular hemoglobin; MCHC, mean corpuscular hemoglobin concentration; PLT, platelet count

Table S5. Histological classification of all pleural mesothelioma cases found in this study.

Group	Animal number	Time of dissection (week)	Condition	Grade	Classification
Low-dose	#L14	53	dead	4	sarcomatoid
	#L28	93	moribund sacrifice	3	sarcomatoid
	#L25	104	terminal sacrifice	1	sarcomatoid
	#L34	104	terminal sacrifice	2	biphasic
High-dose	#H36	79	dead (died from a pituitary tumor)	1	epithelioid
	#H40	80	dead	4	sarcomatoid
	#H16	81	moribund sacrifice	4	biphasic
	#H37	81	moribund sacrifice	3	biphasic
	#H24	82	moribund sacrifice	3	sarcomatoid
	#H26	87	dead	4	sarcomatoid
	#H21	93	moribund sacrifice	4	epithelioid
	#H28	93	moribund sacrifice	4	sarcomatoid
	#H14	104	terminal sacrifice	1	epithelioid
	#H19	104	terminal sacrifice	1	biphasic
	#H31	104	terminal sacrifice	1	epithelioid
	#H38	104	terminal sacrifice	1	sarcomatoid

Grade; 1, focally develop without invasion; 2, disseminate to several regions; 3, distribute over the pleural cavity; 4, form huge masses (more than 10 mm in diameter/thickness)

Table S6. Incidences of other tumors

Organ system	Neoplasm	Control (N=30)	Low-dose (N=29)	High-dose (N=28)
Circulatory system				
Heart	Schwannoma	0	1	0
Hematopoietic system				
Systemic	Large granular lymphocyte leukemia	4	3	4
Digestive system				
Tongue	Carcinoma, squamous cell	0	0	1
Stomach	Hemangioma	0	1	0
Small intestine	Leiomyosarcoma	1	0	0
Reproductive system				
Testis	Interstitial cell tumor, benign	26	24	19
Prostate	Adenoma	2	0	2
Seminal vesicle	Adenoma	1	0	0
Endocrine system				
Pituitary gland	Adenoma, pars distalis	3	5	9
Thyroid gland	Adenoma, C-cell	6	4	2
Adrenal gland	Pheochromocytoma, benign	4	2	1
Pancreatic islet	Adenoma, islet cell	4	3	3
	Carcinoma, islet cell	0	1	0
Nervous system				
Brain	Oligodendroglioma, malignant, high grade	1	0	0
Special sense system				
Harderian gland	Adenocarcinoma	0	1	0
Ear	Neural crest tumor	0	0	1
Zymbal's gland	Carcinoma	0	0	1
Integumentary system				
Skin/Subcutaneous tissue	Fibroma	1	2	1
	Hemangiosarcoma	0	1	1
	Keratoacanthoma	1	0	1
	Osteosarcoma	0	1	0
Tail	Keratoacanthoma	0	1	0
Body cavity				
Peritonium	Mesothelioma, malignant	1	1	1
	Lipoma	1	0	0

Values show the numer of animals with tumors

Table S7. Amounts of MWCNT in the lung and PLF

			week 26	week 52	week 104
Lung	Low-dose	(μg/Lung)	147.1	244.4	920.5
		(μg/g Lung)	123.8	166.6	476.8
	High-dose	(μg/Lung)	728.5	1364.3	3615.4
		(μg/g Lung)	460.3	690.3	1242.0
PLF	Low-dose	(fibers/pleural cavity)	2.70×10^4	4.02×10^4	1.56×10^5
	High-dose	(fibers/pleural cavity)	8.89×10^4	2.82×10^5	1.11×10^6

Mean value (N = 4-5)

Table S8. Mean doses for each administration

	Low-dose group 0.125 mg/kg bw (µg/lung)	High-dose group 0.5 mg/kg bw (µg/lung)		Cumulative dose (µg/lung) Low-dose	Cumulative dose (µg/lung) High-dose
1st	26 ± 1.4	103 ± 6.3			
2nd	33 ± 1.7	134 ± 7.4			
3rd	37 ± 1.8	151 ± 7.6			
4th	40 ± 2.0	162 ± 8.4			
5th	42 ± 2.1	167 ± 9.2			
6th	43 ± 2.2	171 ± 10			
7th	44 ± 2.5	176 ± 9.5	Subtotal at week 26	265	1064
8th	46 ± 2.6	181 ± 10			
9th	47 ± 2.9	186 ± 11			
10th	47 ± 2.9	188 ± 10			
11th	49 ± 2.8	191 ± 11			
12th	49 ± 3.0	193 ± 10			
13th	50 ± 3.0	196 ± 11	Subtotal at week 52	552	2199
14th	50 ± 3.4	195 ± 9.1			
15th	51 ± 3.4	197 ± 10			
16th	51 ± 3.4	200 ± 9.9			
17th	51 ± 3.4	199 ± 10			
18th	51 ± 3.3	199 ± 11			
19th	52 ± 3.4	200 ± 12			
20th	51 ± 3.3	200 ± 11			
21st	52 ± 3.2	198 ± 12			
22nd	51 ± 3.3	199 ± 12			
23rd	51 ± 3.3	198 ± 12			
24th	51 ± 3.4	196 ± 14			
25th	51 ± 3.2	197 ± 12			
26th	50 ± 3.3	196 ± 15	Total at week 104	1215	4770

Left: Mean ± SD of each dose for all 26 administrations

The dose was set corresponding to the individual body weight of each animal for every administration.

Right: cumulative doses at 3-time points
