# Supplementary material for: Two-year intermittent exposure of a multiwalled carbon nanotube by intratracheal instillation induces lung tumors and pleural mesotheliomas in F344 rats
Source: Part Fibre Toxicol. 2022 May 19;19:38. doi: 10.1186/s12989-022-00478-7 (PMC9118836; doi:10.1186/s12989-022-00478-7)
Supplement: Supplementary file 2 — Additional file 2: Fig. S1 Anatomical structure of the mediastinum and histological sample preparations of the pleura. Fig. S2. MWNT-7 fibers piercing the visceral pleura. Fig. S3. Characterization of the bulk MWNT-7: a comparison with Taquann-treated MWNT-7. [file 12989_2022_478_MOESM2_ESM.pdf]

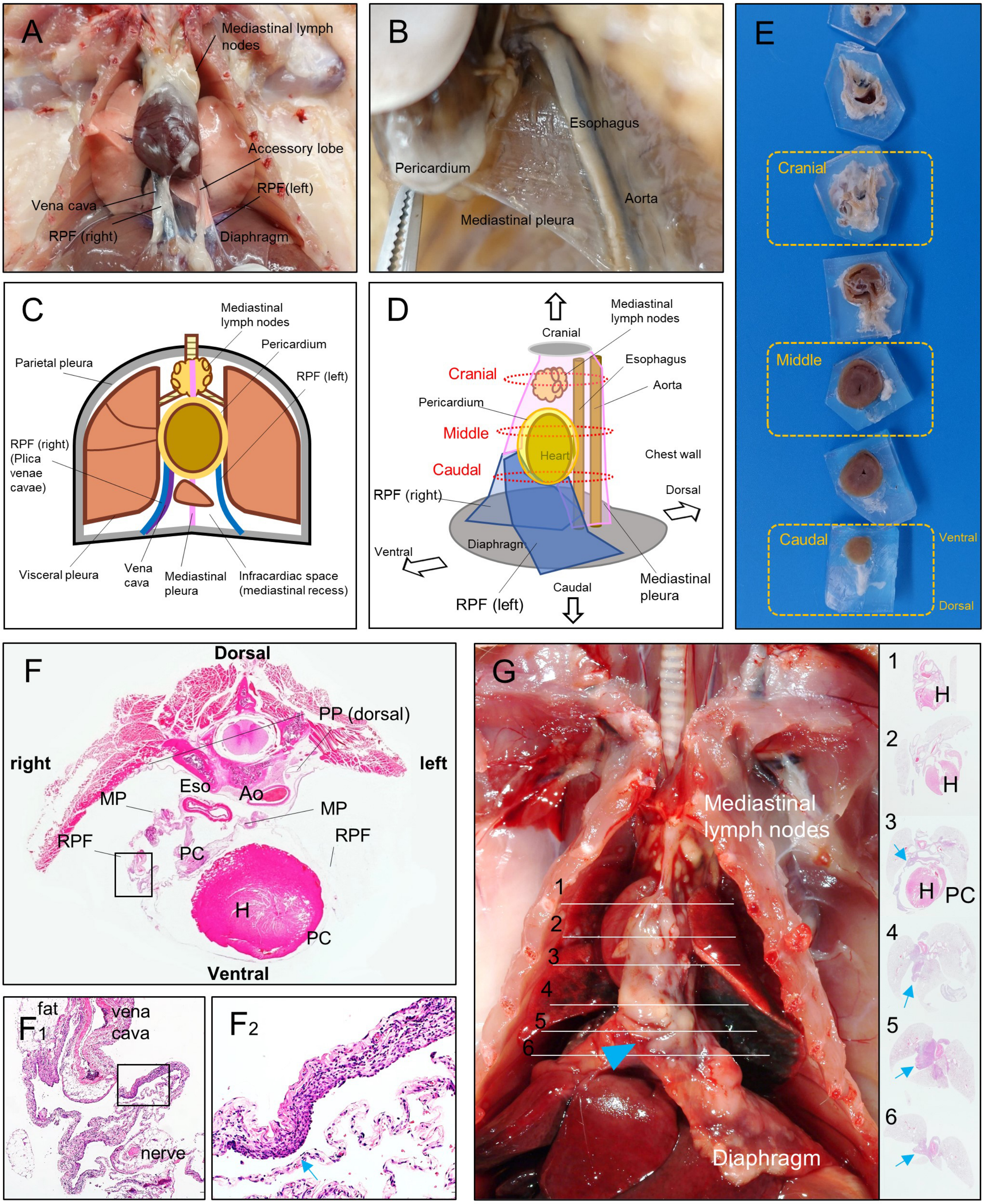

Fig. S1. Anatomical structure of the mediastinum and histological sample preparations of the pleura.

(A) Gross appearance of a thorax of a rat in the control group after removal of the ventral thoracic wall. Retrocardiac pleural folds extend from the pericardium to the diaphragm, covering the accessory lobe. (B) Lateral view of a thorax after removing the lung. The mediastinal pleura extended from the heart to the dorsal thoracic wall, attaching to the esophagus and aorta. Because this rat had a very early phase mesothelioma, the pleura is thickened with foci. (C) Schematic view of the thorax (frontal view). Pericardium, RPF, and diaphragm forms a small space (infracardiac space) where the accessory lobe is encased. Three types of serous membranes in the mediastinum, pericardium (yellow), RPF (blue), and mediastinal pleura (pink) are shown. The right RPF is also called “plica venae cavae.”

Fig. S1.(continued)

(D) Schematic view of the thorax (lateral view). A depiction of the lung is omitted. Three major histological observation levels: cranial, middle, and caudal are shown by dotted red circles. (E) Photograph of a series of cut samples embedded in agar. Three major histological observation levels are shown by dotted rectangles. (F) Loupe image of a caudal-level section. The pleura is thickened and well stained with hematoxylin. (F_1) Histological image of (F). Inflammatory and fibrous changes are visible in the thickened mesothelium. (F_2) High-powered view of (F_1). Mesothelial hyperplasia is seen (blue arrow). (G) Gross appearance of a pleural mesothelioma (case shown in Fig. 3D). The mesothelioma tissue extends along the cranial–caudal axis. The large tumor mass is found at the caudal region (arrowhead). Six sections are shown on right side. The arrows show the mesothelioma. All examples are obtained from the high-dose group, except for (A).

Ao, aorta; Eso, esophagus; H, heart; MP, mediastinal pleura; PC, pericardium; PP, parietal pleura; RPF, retrocardiac pleural fold.

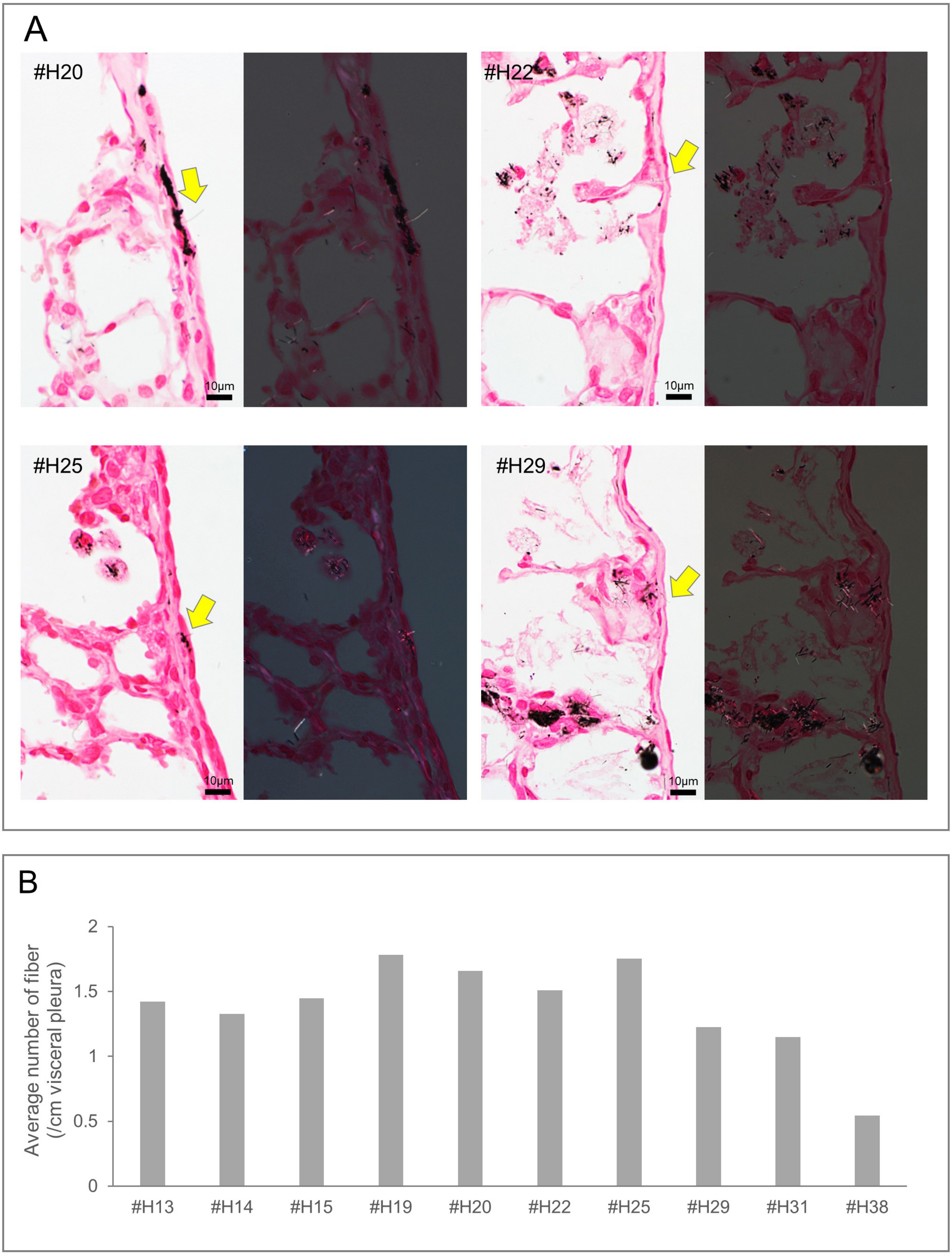

Fig. S2. MWNT-7 fibers piercing the visceral pleura.

(A) Four examples of light microscopic images of the fiber piercing the visceral pleura of a rat in the high-dose group at week 104. Kernechtrot staining. Right side, polarized light microscope image. (B) Numbers of fibers piercing the visceral pleura of 10 animals in the high-dose group at week 104. The average numbers are calculated using 5 sections for each individual.

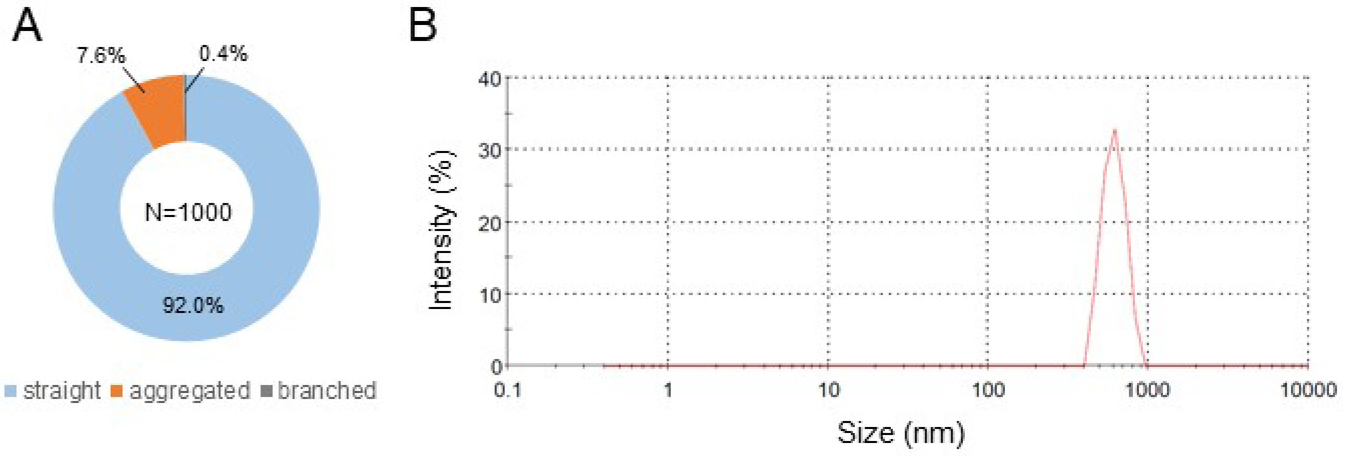

Fig. S3 Characterization of the bulk MWNT-7: a comparison with Taquann-treated MWNT-7 (see Fig.1)
(A) Morphological classification of the individual fibers by SEM. Percentage of aggregated fibers were relatively high. (B) DLS analysis. The secondary particle diameter was 612 nm.
